# From Basic Principles of Protein–Polysaccharide Association to the Rational Design of Thermally Sensitive Materials

**DOI:** 10.1021/acsami.3c12926

**Published:** 2024-02-08

**Authors:** Asaf Rosenberg, Aleksei Solomonov, Hagai Cohen, Dror Eliaz, Israel Kellersztein, Ori Brookstein, Anna Kozell, Linghui Wang, Hanoch Daniel Wagner, Chiara Daraio, Ulyana Shimanovich

**Affiliations:** † Department of Molecular Chemistry and Materials Science, Faculty of Chemistry, Weizmann Institute of Science, Rehovot 7610001, Israel; ‡ Department of Chemical Research Support, Weizmann Institute of Science, Rehovot 7610001, Israel; § Division of Engineering and Applied Science, California Institute of Technology, Pasadena, California 91125, United States

**Keywords:** pectin, silk protein, protein nanofibrils, self-assembly, thermal induced conductivity, biomaterials

## Abstract

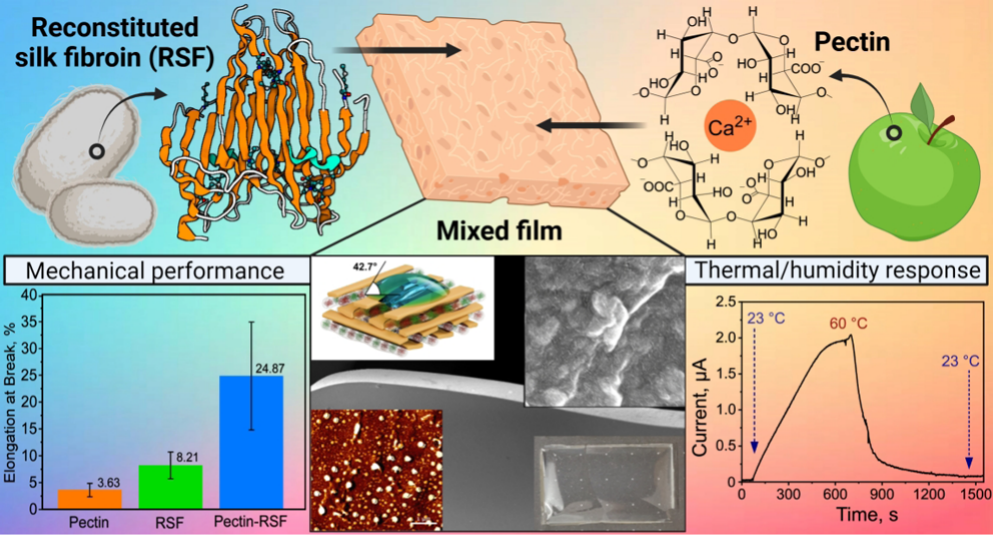

Biology resolves design requirements toward functional materials by creating nanostructured composites, where individual components are combined to maximize the macroscale material performance. A major challenge in utilizing such design principles is the trade-off between the preservation of individual component properties and emerging composite functionalities. Here, polysaccharide pectin and silk fibroin were investigated in their composite form with pectin as a thermal-responsive ion conductor and fibroin with exceptional mechanical strength. We show that segregative phase separation occurs upon mixing, and within a limited compositional range, domains ∼50 nm in size are formed and distributed homogeneously so that decent matrix collective properties are established. The composite is characterized by slight conformational changes in the silk domains, sequestering the hydrogen-bonded β-sheets as well as the emergence of randomized pectin orientations. However, most dominant in the composite’s properties is the introduction of dense domain interfaces, leading to increased hydration, surface hydrophilicity, and increased strain of the composite material. Using controlled surface charging in X-ray photoelectron spectroscopy, we further demonstrate Ca ions (Ca^2+^) diffusion in the pectin domains, with which the fingerprints of interactions at domain interfaces are revealed. Both the thermal response and the electrical conductance were found to be strongly dependent on the degree of composite hydration. Our results provide a fundamental understanding of the role of interfacial interactions and their potential applications in the design of material properties, polysaccharide–protein composites in particular.

## Introduction

Polysaccharide–protein composite materials may offer unique opportunities to realize the material properties beneficial for bio-oriented applications, such as antimicrobial function, biodegradability, response to external stimuli, and thermal and mechanical stability.^1−3^ The physical and structural characteristics of these composites are largely governed by the strength and type of polysaccharide–polysaccharide, protein–protein, and polysaccharide–protein interactions. Such interactions, in general, could be either associative or segregative.^1^ Associative interactions are formed when two different biopolymeric components are attracted to each other, whereas segregative interactions occur when they are mutually repelled. Although polysaccharide–protein complexes have been extensively studied, especially for food-manufacturing purposes,^4^ our understanding of how their material properties evolve in nature,^1,5,6^ through controlled sets of molecular and nanoscale interactions, is still limited. We have not yet elucidated, for example, the thermal response of the plant polysaccharide pectin, which varies its electrical conductivity in response to small temperature changes^7−9^ or the ability to modulate the mechanical properties of silk fibroin fibers.^10−12^ In this work, we create composite materials by combining pectin and silk fibroin and study their interactions. We explain how these interactions guide their supramolecular assembly pathways as well as the thermal response and the mechanical performance of their final composite material.

Many natural biopolymers exhibit unique, often unexpected physical properties, especially when taking into account the high instability of the monomeric protein or the polysaccharide building blocks. The plant polysaccharide pectin, the structural component in the primary cell walls of terrestrial plants,^13,14^ has the unique ability to conduct electrical current that is highly sensitive to temperature changes.^15−17^ Pectins are rich in galacturonic acid, which influences properties such as porosity, surface charge, pH, and ion balance. Therefore, pectins are critical ingredients in the ion-transport mechanisms within cell walls.^18−20^ Natural pectins contain multiple negatively charged saccharide components that can bind cations, in particular, Ca ions (Ca^2+^), and thereby form a highly ordered, carboxylate-rich, cross-linked “egg-box” structure, which confers electrical conductivity via the diffusion of Ca ions (Ca^2+^) along the egg-box.^21,22^ However, the study of these electrical properties of pectins has thus far focused on end-point material performance, leaving our understanding of the ionic conduction mechanism, as well as its dependence on thermal and humidity factors, limited.

Noted for its unique physical properties,^23,24^ the silk fibroin protein is produced by a variety of insects and arachnids, including silkworms and spiders, which spin it into micron-scale fibers with exceptional mechanical strength and extensibility (and consequently, toughness).^25,26^ At the molecular level, the self-assembly process of many proteins,^27,28^ especially the fibrous assembly of silk, is similar to that of functional amyloids. Under certain conditions, which include changes in pH, ionic strength, or shear forces (created during the spinning process by the elongational flow), the protein loses its native conformation (via partial or full misfolding), and instead adopts a new β-sheet-rich conformation that is stabilized via a continuous hydrogen-bonded network.^29,30^ Interestingly, recent studies show that controlling the structural composition of silk-based materials, specifically the relative ratio between the ordered β-sheet-rich and the disordered random coil or the α-helix conformation, enables modulation of the materials’ mechanical properties.^31^

The combination of pectin and silk fibroin is particularly interesting in designing new biocomposite materials with multifunctional properties. Both components, silk fibroin protein and pectin polysaccharide, are abundant, cheap, and available in industrial quantities. The ultimate aim of specifically combining these two materials is to create a biocompatible and stable composite material with tunable multifunctional properties. The ability to control silk protein fibrillation and therefore, to design and program the mechanical properties of the final composite, on the one hand, together with the tunable thermal responsivity of pectin, fulfills our demand toward achieving desired characteristics of their composite material. In particular, the mechanical resistance offered by silk, combined with the thermal sensitivity of pectin, opens up new opportunities for organic composite materials used as wearable medical sensors and flexible electronics.^32−36^

Here, we show how the weak molecular-level interactions between pectin and silk fibroin modify their natural propensity to form mesoscale supramolecular assemblies and thus affect the material properties of their composite. Our experiments consistently indicate that mixing silk and pectin at comparable ratios (see the Experimental Section) generally leads to spontaneous phase separation, which prevents any composite formation. Yet, a “narrow window” of silk/pectin ratios was found in which the phase separation takes place at the nanoscale, thus giving rise to markedly different and attractive material properties. Based on standard electrical measurements, combined with controlled surface charging (CSC) in X-ray photoelectron spectroscopy (XPS),^37^ we show that silk fibroin can enhance considerably the dependence of Ca ions (Ca^2+^) diffusion on humidity. This effect is enabled through segregative phase separation (at the nanoscale) between pectin and fibroin, thus introducing restrictions to the allowed orientations of pectin molecules. In agreement with X-ray diffraction (XRD) and wide-angle X-ray diffraction (WAXS) reports,^11^ the generation of small domains further triggers limited conformational changes in fibroin, which we resolved by Fourier transform infrared (FTIR) spectroscopy. As a result, properties are modified to enhance the “water-holding” capacity, hydrophilicity, ion conductivity, and mechanical strain, which consequently affect the temperature dependence of the electrical conductivity. Consequently, our results suggest that the controlled modulation of polysaccharide–protein phase separation can be utilized as a promising concept for the design and manipulation of structural and physical characteristics in composite biomaterials.

## Results and Discussion

### Supramolecular Organizational Differences between Pectin, Fibroin, and Pectin–Fibroin Thin Films

Polysaccharide–protein composite materials were prepared by mixing aqueous pectin and aqueous silk fibroin at different ratios (see the Experimental Section), which were then cross-linked by Ca ions (Ca^2+^) in a CaCl_2_ solution. A detailed exploration of the process using varied pectin/RSF ratios showed that homogeneous mixtures could be formed with (before mixing) ∼1% pectin and ∼6% fibroin. On the one hand, when pectin and RSF were mixed at comparable molar ratios, namely, pectin/fibroin ratios larger than 1:6, spontaneous phase separation was observed. On the other hand, for concentration ratios below 1:6, loss of conductance and thermal responsivity was detected. Therefore, focus is put here on mixtures of the 1:6 concentration ratio. Composite films were thus formed (see the Experimental Section), exhibiting which were then cross-linked by Ca ions (Ca^2+^). Commonly for pectin monomers, cross-linking via Ca ions (Ca^2+^) leads to the formation of a supramolecular structure in the form of highly ordered channels with an egg-box shape. The internal part of the channels is rich in carboxylate COO^–^ groups. Such a structural organization can support Ca ion (Ca^2+^) and proton diffusion along the channels, which would also regulate the thermal response observed in earlier studies.^7,8^

**Figure 1:**
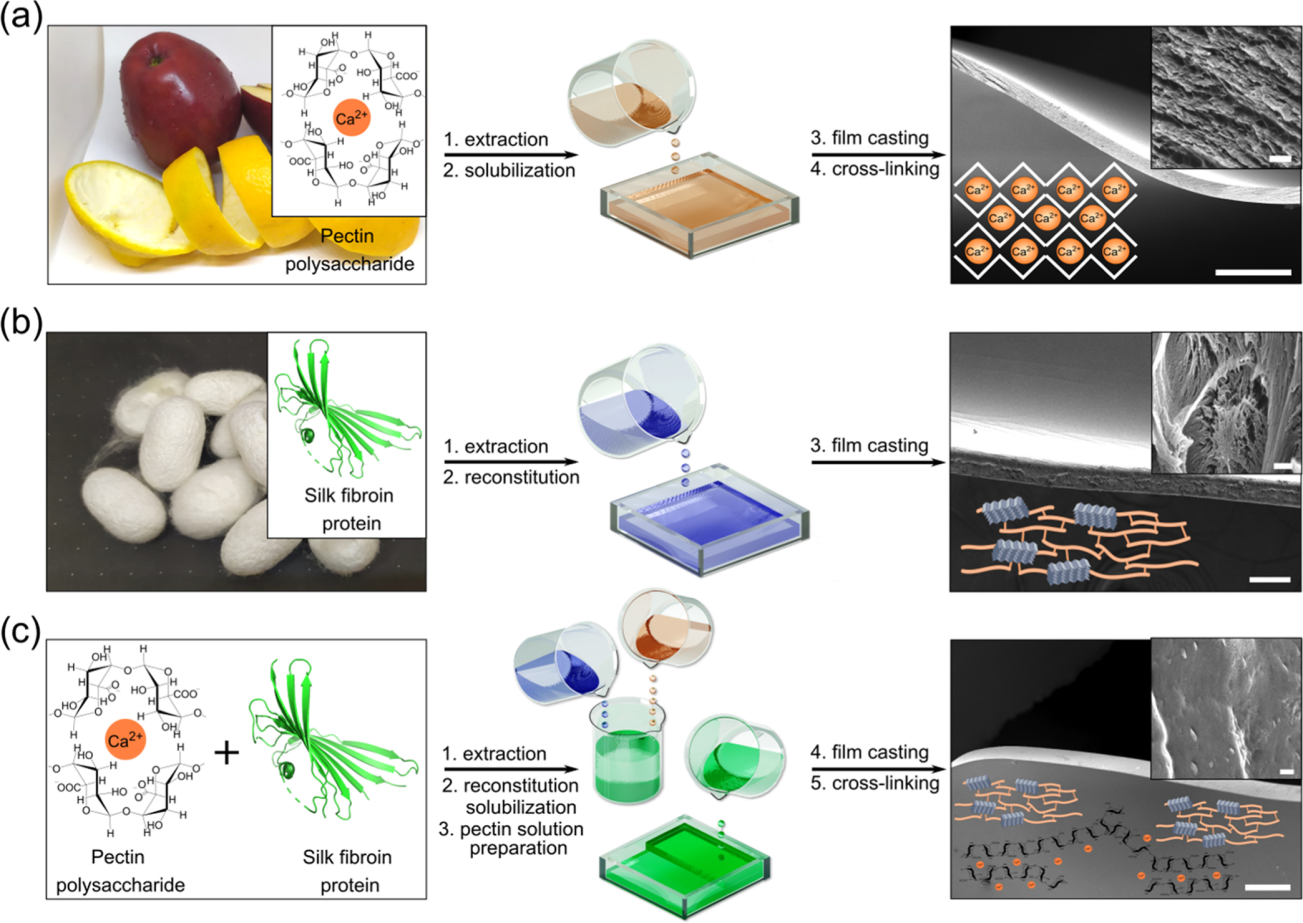
Preparation of (a) pectin, (b) silk fibroin (fragment, N-terminal domain, PDB ID: 3UA0), and (c) pectin–silk fibroin composite thin films. Left: pictures of the source of (a) the pectin polysaccharide (i.e., fruit) and (b) fibroin protein (i.e., *B. mori* silkworm cocoons), with the insets showing the chemical structure of the pectin polysaccharide biopolymer (a) and molecular structure of fibroin chain (b). (c) Molecular structure of pectin and silk fibroin biopolymers in their composite form. Middle: graphical demonstration of each film type’s casting procedure. Right: SEM images of the corresponding films and a graphical demonstration of the molecular assembly, with each inset showing an enlarged image of the film morphology. The scale bar for the SEM images is 100 μm, and that for the inset images is 400 nm. Cartoon schematic insets for SEM images (bottom left): (a) pectin chain with Ca ions (Ca^2+^), (b) a fibrillar silk β-sheet-rich structure consisting of silk monomers (image was created with BioRender.com), and (c) a pectin–silk hybrid composite.

We compared the structural and physical characteristics of the following films: Ca ions (Ca^2+^)-cross-linked pectin-only (hereafter referred to as “pectin” films), reconstituted silk fibroin (RSF) only (hereafter referred to as “silk fibroin” or “RSF” films) and Ca ions (Ca^2+^)-cross-linked pectin–fibroin composites (hereafter referred to as “pectin–silk fibroin composite” films or “composite” films) (see details in the Experimental Section and in Supporting Information, Figure S1). Scanning electron microscopy (SEM) of the resulting films, 15–40 μm thick, revealed morphological and micrometer-scale organizational differences (Figure 1a–c). The pectin films featured a highly ordered layered lamella-like structure (Figure 1a, right), whereas the silk fibroin films exhibited a nanofibrillar morphological organization (Figure 1b, right). In turn, the composite films were characterized by nonhomogeneous morphology (Figure 1c, right), with no evidence of an ordered structural organization. These morphological changes emerged from the introduction of domain interfaces with weak molecular interactions between the polysaccharide pectin and the silk fibroin protein.

### Analysis of Structural Characteristics by Using FTIR

**Figure 2:**
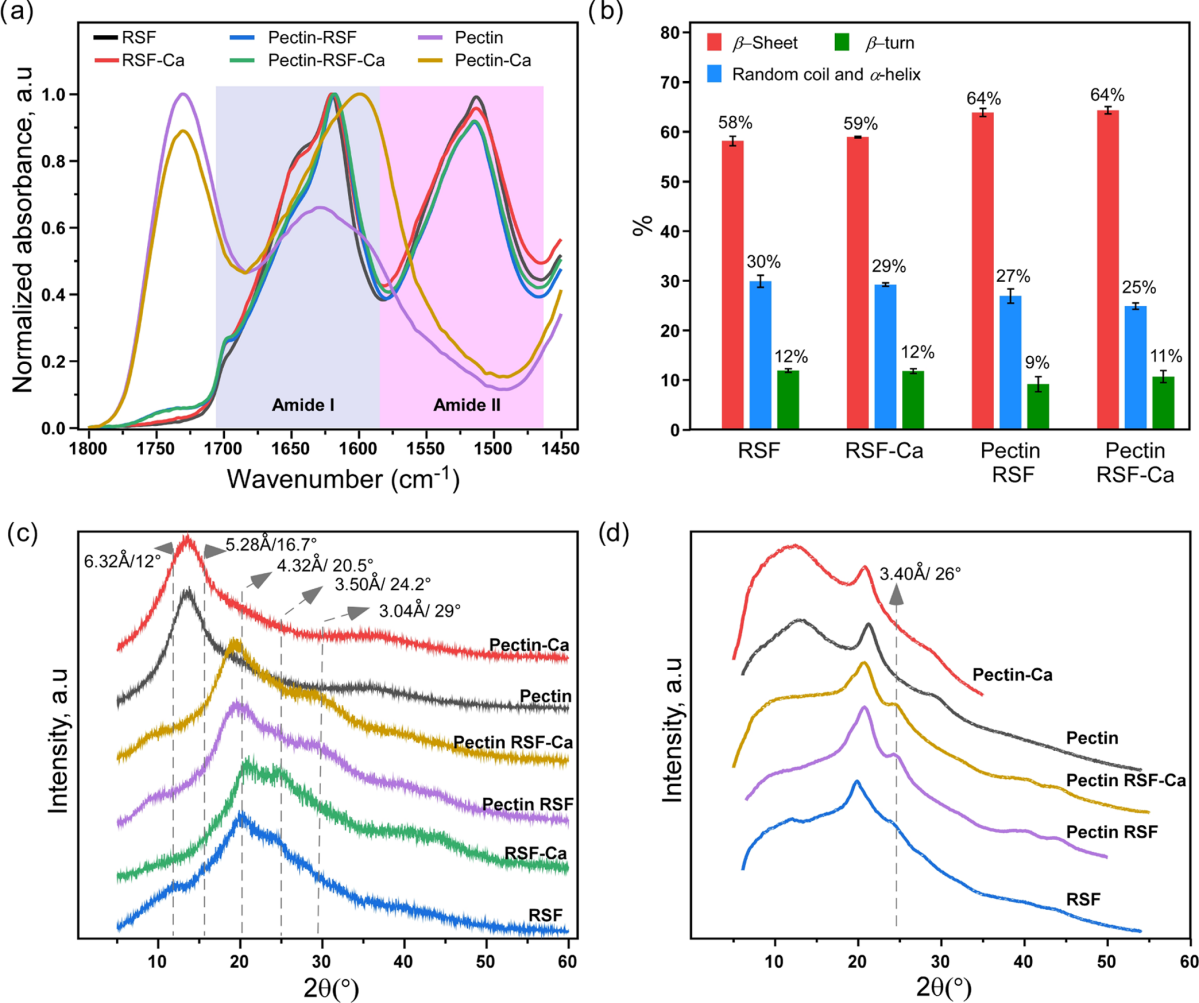
(a) Amide I FTIR spectra of silk fibroin (RSF), pectin, and pectin–fibroin composite films (RSF Pectin). (b) Comparative analysis of the secondary structure of (a) with the band positions of the β-sheets at 1610–1635 cm^–1^, antiparallel β-sheets at 1690–1705 cm^–1^, random coil and α-helixes at 1635–1665 cm^–1^, and β-turns at 1665–1690 cm^–1^. (c) XRD images of the studied films. Peak positions and corresponding distances are indicated. (d) WAXS of representative films.

Insight into the molecular-scale (structural) organization of the pectin–fibroin composite is gained from the Fourier transform infrared spectroscopy (FTIR) analysis of the pectin, silk fibroin, and composite films (Figures 2a,b and S2). First, we inspected the changes in silk protein conformations in the presence of a pectin biopolymer. In general, the vibrational spectra of proteins are characterized by two major bands, amide I (1600–1700 cm^–1^) and amide II (1480–1600 cm^–1^), which correspond to C=O and NH bend/CH stretching, respectively,^38^ as well as by amide A bands (>3000 cm^–1^). The amide I region is commonly used to characterize the secondary structure of proteins, *e.g.*, their intermolecular β-sheet (1610–1635 cm^–1^), random coil/α-helix (1635–1665 cm^–1^), β-turn (1665–1690 cm^–1^), and antiparallel β-sheets (1690–1705 cm^–1^).^39^ The comparative FTIR analysis revealed a small increase (∼5%) in the fraction of β-sheet conformations in the pectin–silk fibroin composite film, compared with the silk fibroin film (Figure 2b). Moreover, the composite material featured aggregative β-sheet content, with characteristic peaks at 1620 and 1700 cm^–1^, which is in agreement with literature reports of an aggregative antiparallel β-sheet organization, as well as with our XPS analysis (see the Supporting Information for a description of the XPS evaluation of hydrogen bonds and the related Figure S3).^31,39,40^ A small pectin signal that overlaps with the amide I band is already accounted for in the present analysis. Interestingly, the silk fibroin films featured a lower fraction of β-sheets compared with the composite material, indicating the structural reorganization-inducing effect of pectin on the silk protein.

To better understand the structural changes of constituents in the composite material, we performed sets of XRD and WAXS measurements (Figure 2c,d). The comparison between these two diffraction configurations provides orientational information because XRD selectively depicts the *k*_z_ information (hence, the vertical *d*-spacings), whereas WAXS probes mixed in-plane and out-of-plane *k*-vectors. The pure pectin films are well oriented parallel to the film surface, as intuitively expected from this material; hence, the 29° peak in Figure 2d, associated with an in-plane periodicity, is absent from the pectin XRD patterns in Figure 2c. Complementarily, the pectin 13.4° peak, attributed to its vertical interplane spacings, is far more dominant in the Figure 2c patterns, compared with Figure 2d.

Remarkably, the corresponding diffraction peaks in the composite samples present significant changes, indicating that the pectin domains do not preserve a common orientation when incorporated between the RSF domains. In particular, the 13.4° peak is drastically suppressed in the XRD of the composite, which suggests that besides orientational changes, the limited size of the pectin domains allows only a short-range order along their “out-of-plane” direction.

The pure RSF patterns do not exhibit a high orientational order in the first place, as can be deduced from the comparison between their XRD and WAXS patterns. However, upon mixing with pectin, an interesting feature is observed in the RSF peak at ∼20°, attributed to the β-sheet-related 4.3 Å spacing. Compared to the pure RSF, this peak shifts to a slightly lower angle (note the somewhat confusing overlap with a pectin peak at a slightly larger angle). This RSF-related peak shift indicates a slight increase in the β-sheet spacing when the long-range formation of β-sheets is interrupted.

Combined with the FTIR molecular-scale structural data, our analysis reveals that the interaction between the polysaccharide and protein biopolymers, which is realized via H-bonds, leads to conformational changes in the protein molecules. Results are consistent with the segregative type of interconstituent interactions, where phase separation leads to the formation of small homogeneous domains. These findings naturally raise the question of whether the noted changes in molecular interactions also impact the biopolymer organization at the mesoscopic scale and further change the microscopic properties of the biopolymers in the composite material. To address these questions, we probed the mesoscopic behavior of the biopolymers by evaluating the rheology of the precursor solutions of pectin, silk fibroin, and a pectin–fibroin mixture.

### Studying the Mesoscopic-Scale Behavior of Pectin–Fibroin Biopolymers in Their Composite Form

Pronounced differences were observed in the fluid behavior of the one-component solutions compared to their mixture (Figure S4a–e). The aqueous solutions of both pectin and silk fibroin (Figure S4a,b,d,e) exhibited a shear-thinning behavior (the fluid flow behavior was examined under an acting shear stress at a rate of 5–2000 s^–1^ for 8 min, see details in the Experimental Section). Although the observed shear-thinning fluid behavior of the pectin solution was predictable due to the intrinsic propensity of the polysaccharide molecules to form a layered lamellar structure, the shear-thinning behavior of the silk solution was unforeseen. However, taking into account that silk fibroin is highly sensitive to shear stress, we attribute the shear-thinning fluid behavior to changes in the silk protein’s conformation as a function of both the applied shear and pH.^40−42^ Generally, the entire sequence of silk fibroin is classified into three main regions: the repetitive hydrophobic motif with an isoelectric point of pI = 3.8, the negatively charged hydrophilic domains at the N-terminus (pI = 4.6), and the positively charged ones at the C-terminus (pI = 10.5).^43^ The freshly prepared degummed silk fibroin solution exhibited a pH of 9.4. At this pH, all of the charged amino acids, except for those at the C-terminus, are likely to be negatively charged. Indeed, the ζ-potential analysis revealed that silk fibroin carries a negative charge corresponding to an electrostatic potential of −8.8 mV (Figure S5). Such a charge distribution creates a repulsive interaction between the residues that forces the entire protein to adopt an elongated molecular conformation, which contributes to the shear-thinning fluid behavior.^40,44^ Interestingly, in the pectin/fibroin mixture, the shear-thinning effect decreases. This effect points to a possible interaction between pectin and silk fibroin. The pH of the pectin–silk fibroin mixture is 4.6, which is lower than that of the silk fibroin solution (pH = 9.4) and higher than that of the pectin solution (pH = 3.2). Lowering the pH leads to the suppression of dominant repulsive interactions, a phenomenon that promotes a less extended, more compact conformation. This observation is in good agreement with our XPS and FTIR results, which indicate the formation of a larger fraction of β-sheet protein conformations upon interaction of silk fibroin with pectin.

### Macroscale Characteristics of Pectin–Fibroin Materials

**Figure 3:**
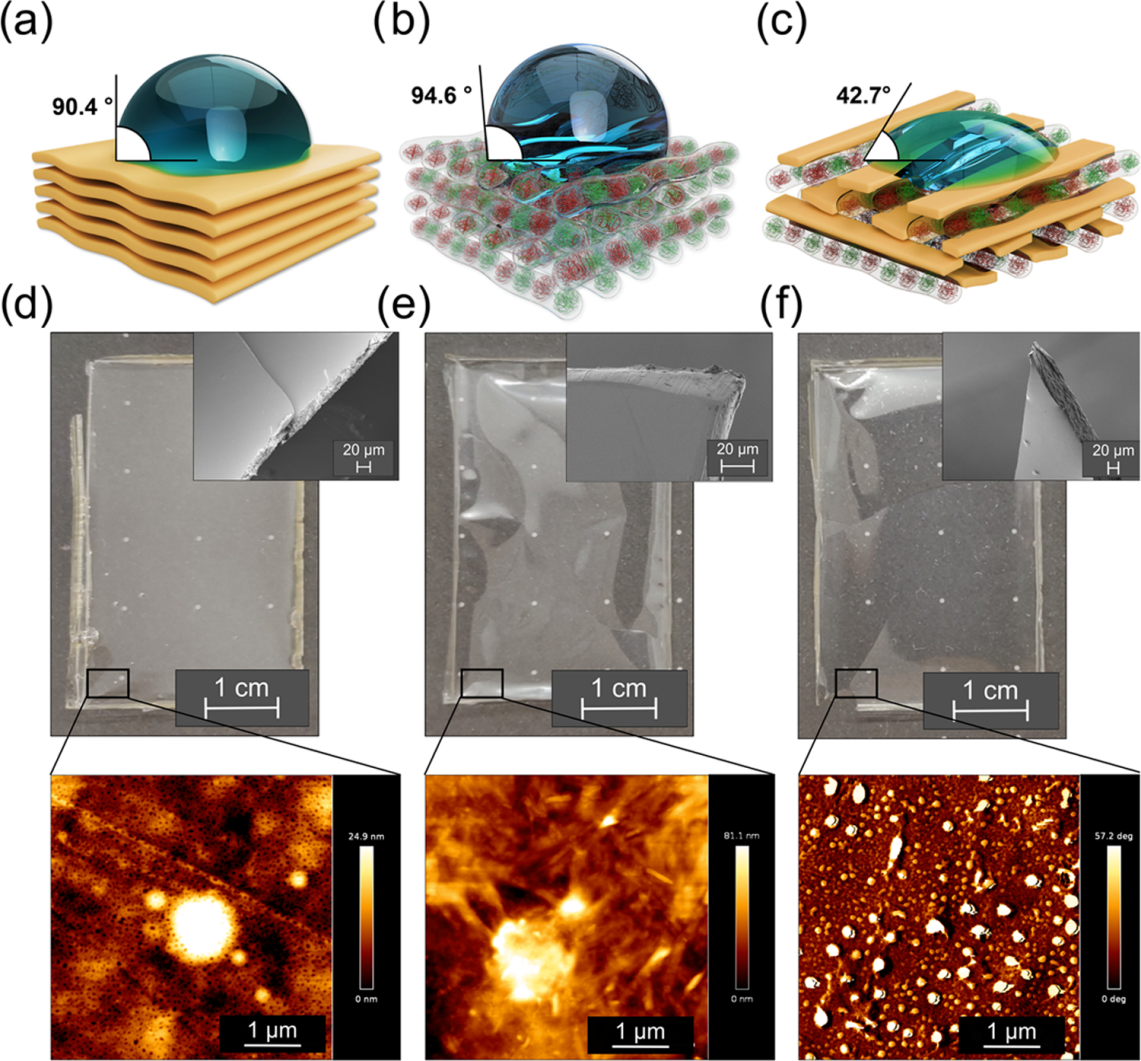
(a–c) Contact angle measurements for the (a) pectin film—90.4°, (b) RSF film—94.6°, and (c) pectin–RSF composite film—42.7° (see the original data in Supporting Information, Figure S6), images were created with Autodesk Fusion 360; (d–f) Pictures of the (d) pectin, (e) fibroin (RSF), and (f) pectin–RSF composite films with an SEM image as an inset and an atomic force microscopy image of the respective film below each SEM image. The scale bars are 1 cm in the optical images, 20 μm in the SEM images (insets), and 1 μm in the AFM images.

In terms of interfacial characteristics, both the pectin and silk fibroin films formed relatively hydrophobic surfaces with contact angles (CA) of θ = 90.4 and 94.6°, respectively (Figures 3a,b and S6). The surface of the composite film exhibited a marked increase in hydrophilicity with a contact angle of θ = 42.7° (Figures 3c and S6). This observation might arise from two interconnected aspects of the separated pectin and silk domains formation: First, upon growth at domains of limited size, silk protein tends to sequester its hydrophobic sequence motifs into the inner part of the domain, thus localizing hydrophilic regions toward the interface. Second, the newly formed domain interfaces act as water trappers, thus increasing the water-holding capacity of the composite material; as indeed observed experimentally by the increased hydrophilicity of the composite. Reorientation of the silk motifs is consistent with the existing literature reports.^31^ We observed that when fibroin protein is destabilized (either by changes in pH or by exposure to ions or the creation of a crowded environment) it triggers the formation of nanoscale compartments, where the hydrophilic motifs are oriented toward the interface and sequester hydrophobic repetitive sequences.^31^ More specifically, the silk fibroin protein is generally composed of repetitive hydrophobic sequences (GAGAGS) and C- and N-terminal hydrophilic sequences. At both macro- and micro scales, all of the prepared materials represent transparent films, and according to the SEM and AFM images, they are smooth and uniform (Figure 3d–f), showing no surface peculiarities, whereas with RSF, a fibrillar structure is observed (Figure 3f). Finally, the Pectin–RSF composite shows distinct phase separation features (Figure 3g) in the presence of calcium ions. A similar effect of phase separation, though of a different nature, was observed upon silk fibroin self-assembly in the presence of calcium and hydrophosphate ions.^45^

Upon interaction with the pectin polysaccharide, the hydrophobic regions of the protein adopt a β-sheet conformation. Such structural changes in protein are also accompanied by the sequestering of newly formed β sheets into the core and the reorientation of hydrophilic regions toward the interface; this is in good agreement with our structural (FTIR) and the rheological findings. These structural changes promote increased surface wetting (Figure S6).

We further evaluated the water content and the “water-holding” property of the films via a thermogravimetric analysis (TGA) (Figure S7a–c). The major peaks measured are summarized in Figure S7d and are assigned to the following processes: (1) water loss, (2) breakage of the H-bonded network, (3) disassembly of the protein/polysaccharide backbone structure, and, finally, material failure. In all stages, the composite peaks appeared at higher temperature values than those of the one-component films. In particular, water loss in the pectin and silk fibroin films was at 65 and 70 °C, respectively, whereas in the composite film, it took place at 110 °C. H-bond breakage occurred at 212 and 208 °C in the pectin and silk fibroin films, respectively, whereas in the composite film, this peak appeared at 250 °C. The pectin and silk fibroin film backbone decompositions were recorded at 236 and 279 °C, whereas the decomposition of the composite film was at 283 °C. The final material decompositions of all three films occurred at similar temperature values.^46,47^ This observation confirms that the interaction between two components, pectin and silk fibroin, allows overall stability of the composite material and, in particular, enhances its “water-holding” capability. Notably, it is the mixture of small domains that allows “stress absorbance” at the boundaries.

### Investigation of the Ionic Conduction in the Composite Films

Further information on the composite biopolymer-based materials was obtained by inspecting the electrical conductance under controlled conditions for the mixed, one-component pectin, and the silk fibroin films. Literature reports^7,8^ have already observed that the interaction of Ca ions (Ca^2+^) with the COO^–^ groups in pectin is noncovalent and therefore varies dynamically in response to temperature and humidity changes. To start with, both the temperature increase and humidity were found to enhance proton conductivity. In the present work, we investigated the role of similar effects on the diffusion of the Ca ions (Ca^2+^). In the present work, the interaction with silk is investigated by comparing the macroscopic characteristics of the three biopolymer-based materials: (i) the cross-linked pectin films, (ii) the composite pectin–fibroin films, and (iii) the fibroin films (as well as silk fibroin films doped with Ca ions (Ca^2+^), as a control). Interestingly, the electrical conductance of Ca ions (Ca^2+^) in the mixed material exhibited a marked increase in sensitivity to humidity caused by the presence of silk. The reader is further referred to complementary experiments shown in Figure S14 and summarized in Table S2, which provide quantitative details on the Ca vs proton ion conduction characteristics.

**Figure 4:**
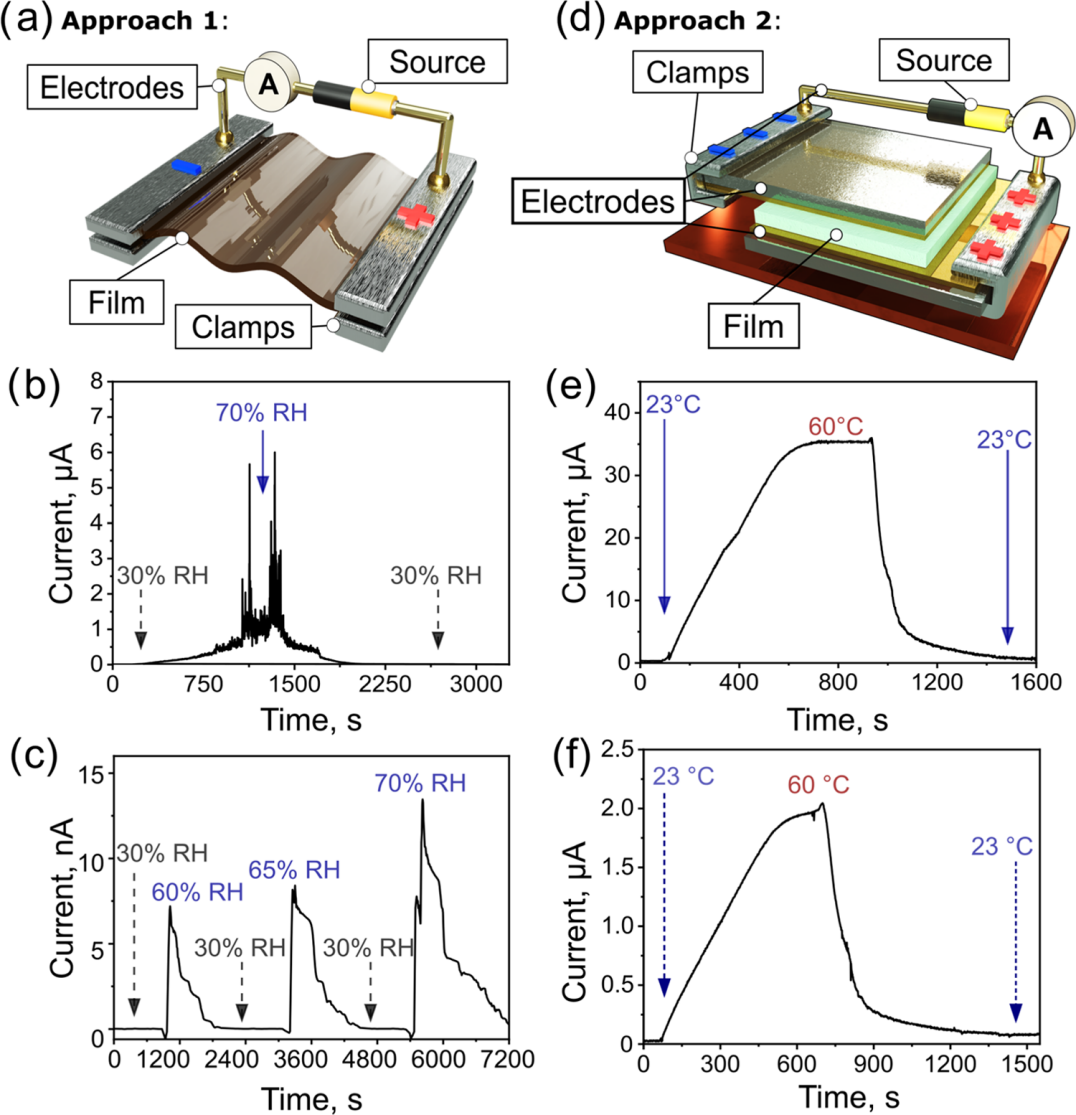
(a) Schematic representation of the Approach 1 experimental setup for evaluating the electrical properties of the studied films as a function of the humidity changes. The setup consists of electrodes, clamps for film fixation, and a source. (b) Changes in the pectin film’s current response due to humidity changes (from 30 to 70% and then back to 30%) as a function of time at 23 °C. (c) Changes in the composite film’s current response due to humidity changes (humidity values: 30, 60, 30, 65, 30, and 70%) as a function of time at 20 °C. (d) Schematic representation of the Approach 2 experimental setup for evaluating the electrical properties of the studied films as a function of temperature changes. The setup consists of contact double-layered electrodes on the top and bottom, which enable uniform heating of the sample, clamps for the films’ fixation, and a source (see the Experimental Section). (e, f) Changes in the (e) pectin film’s and (f) the composite film’s current in response to the changes in temperature (from 23 to 60 °C and then back to 23 °C) as a function of time at a relative humidity of 30–33%. Images (a) and (d) were created with Autodesk AutoCAD.

Two approaches for evaluating the electrical properties of the three films are described here. In the first (Approach 1), we determined the films’ conductivity as a function of humidity changes (Figure 4a). To this end, the prepared films were placed on a microscope glass and fixed to two electrodes, as shown in Figures 4a, S8a, and S12a. The samples were then subjected to slow and rapid changes in relative humidity (between 30 and 70–95%). We found that the current increased under increased humidity conditions for all four films (Figures 4b,c, S9, S10, S12b,c, and S13). All of the investigated films also exhibited reproducible cycle changes in response to the respective humidity change cycles. This indicates that the process of hydration/dehydration (i.e., moisture adsorption) is reversible for all of the samples. Further experiments that successfully resolved the proton vs the Ca ion (Ca^2+^) conduction contributions are described in the Supporting Information file.

In the second approach (Approach 2), using a different experimental configuration, we probed the electrical properties of the films as a function of the temperature variations. For this purpose, the film was placed in between a pair of iridium (or gold) square electrodes (Figures 4d and S8b–d), 5 mm × 5 mm in size, prepared using lithography and sputtering on a silicon wafer and a microscope glass. Next, the sandwich assembly was placed on a hot plate surface located within a probe station, and the thin films’ resistance was monitored over time as a function of temperature (Figures 4e,f and S11). Samples were subjected to slow temperature variations, between 23 and 60 °C, during one cycle of ramping temperatures up and a slow cooldown to ambient conditions (see the Experimental Section). All measurements were performed at thermal equilibrium, showing that the resistance decreases with increasing temperature. The stability of the material for extended periods at elevated temperatures under a constant humidity of 40% was established by means of holding the temperature constant at 60 °C for approximately 10 min before cooling. Under these conditions, the conductivity of the sample remained constant. Interestingly, when humidity was not maintained constant, a stronger decrease in the current was observed, a phenomenon that suggests sample dehydration.

Analysis of the electrical characteristics under various conditions (Table S1), revealed an equilibrium at 20–23 °C and 30% humidity. It indicates that the conductivity of the pectin film, as expected, is the highest among all of the studied films, whereas the conductivity of RSF is the lowest. Doping the RSF film with Ca ions (Ca^2+^) increases its conductivity ca. 1.5- to 2-fold; however, compared to the pectin film conductivity, such an improvement is negligible. The composite film’s conductivity was ∼55-fold higher than that of the RSF film and ∼14-fold lower than that of the pectin film. Increasing the humidity from 30 to 70% led to an expected increase in the conductivity of all of the materials. We thus found for the pectin, composite, and Ca ions (Ca^2+^)-doped RSF films, respectively, ca. 10-, 54-, and 7- to 8-fold increase in conductivity. Consequently, the effect reveals the dominant role of transport along domain interfaces. Independent indications of the accumulation of water molecules at the pectin–RSF interface support this proposal. Finally, faster Ca ion (Ca^2+^) conduction is enabled under increased humidity. A similar mechanism associated with Ca-transport acceleration has already been suggested in earlier literature;^7^ however, the present experiments provide a more direct evaluation of this mechanism.

Increasing the temperature to 60 °C, beyond a small rise in humidity, also increased the conductivity: ∼107-fold in pectin film, ∼74-fold in the composite film, and ∼3-fold in the RSF film, whereas in the Ca-doped RSF film, an ∼15-fold increase was observed, demonstrating that the Ca-doping accounts for the major conductivity increase upon temperature elevation. The ratio between the current measured at 23 °C and that at 60 °C was termed as the “thermal potential” of the film, as can be seen in Table S1. The fibroin component had a moderating impact on the thermal potential of the pectin–silk fibroin composite film. Thus, the dual presence of pectin and Ca ions (Ca^2+^) in the pectin–RSF composite makes it significantly more conductive, which can be exploited to connect the samples to an external circuit.

### Mechanistic Insights into Ca^2+^-Based Ionic Conduction by Using XPS

The ionic conductivity in biomaterials, which are assembled from natural biopolymers, can in practice be analyzed by tracking the diffusion of Ca ions (Ca^2+^) under intentionally applied electric fields during the XPS measurement. A technique for controlled surface charging (CSC) has already demonstrated the unique analytical capabilities of the XPS tool,^37^ including the deintercalation of alkaline ions from layered compounds.^48^ Here, vertical diffusion should spectrally show up in the intensity of Ca, relative to the other elemental signals and, thus, provide insights into the interactions of Ca^2+^ ions with their environment, including the electronegative carboxylate groups (COO^–^).^31,38^

**Figure 5:**
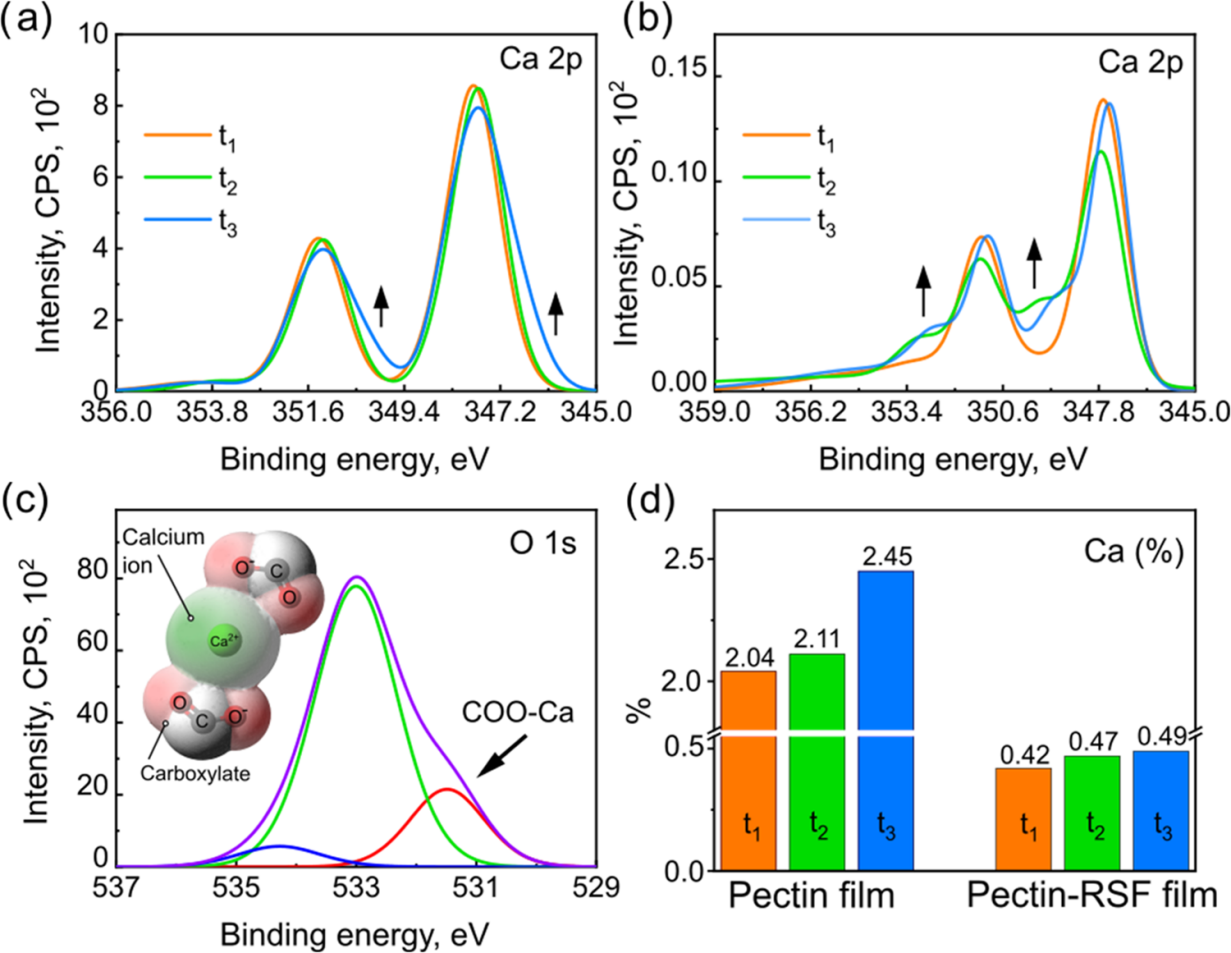
(a–c) Time-dependent X-ray photoelectron spectroscopy (XPS) of the polysaccharide pectin and its composite with silk fibroin protein. (a, b) XPS spectra, normalized in order to visually emphasize changes in the line shape of (a) Ca in the pectin-only film and (b) Ca in the pectin–fibroin composite films. (c) Curve fitting for the O 1s XPS line in the pectin-Ca^2+^ film (O 1s, purple) showing a shoulder attributed to the egg-box oxygen atoms (COO-Ca, 531.4 eV, red) and a graphical representation of the electron density distribution around the two interacting groups: an electropositively charged calcium ion and two electronegative carboxylate groups (inset). (d) Quantitative evaluation of the beam-induced changes in the absolute atomic concentration of Ca, as revealed by XPS. *t*_1_, *t*_2_, and *t*_3_ in (a), (b), and (d) are the time points during a long experiment, 24 h in total: 12 (*t*_1_), 60 (*t*_2_), and 120 (*t*_3_) min. All time values refer to the beginning of the sample exposure to the X-ray + eFG irradiation, normalized to 75 W in the X-ray source power and averaged because of delays between C, O, and Ca, as dictated by serial scans. Differences between the pectin and the mixed sample should be noted, resulting from the longer scans of the Ca 2p line, which were essential in the case of a mixed sample. Note also the finite loss of OH groups and the related changes in the O 1s and C 1s spectra, described in Figure S2 in the Supporting Information.

We, therefore, stabilized the XPS electron flood gun (eFG) conditions such as to get fixed surface (negative) charging, and under these conditions, we compared XPS data that were recorded at different times throughout the experiment. This procedure was repeated for samples composed of (i) Ca ions (Ca^2+^)-cross-linked pectin films, (ii) fibroin films, (iii) fibroin films doped with Ca ions (Ca^2+^), and (iv) Ca ions (Ca^2+^)-cross-linked pectin–fibroin composite films (see the sample preparation procedures in the Experimental Section and the detailed discussion in the Supporting Information). Representative results are summarized in Figure 5a–c and in Figure S3. Long-time exposures of the pectin films to simultaneous irradiation by the X-ray beam and the eFG indeed resulted in an increased intensity of the Ca peaks. Considering the relative intensity changes, the Ca increase was much larger than all of the other elements, indicating an outward diffusion of Ca ions (Ca^2+^) from the bulk to the surface (see Figures 5a–c and S16).

Notably, the average binding energies of the Ca 2p doublet shifted to lower values (Figure 5a), exhibiting a shoulder of partially reduced Ca. This effect is associated with a limited effect of the free eFG electrons on the Ca ions (Ca^2+^) that diffused to the top surface. In fact, a notable point here concerns the fact that the diffusion process was not remarkably high, suggesting that the electric field could indeed activate the diffusion but was insufficient to enable a continued process. Recalling the pronounced humidity role observed in the ambient electrical measurements, Figure 3, the XPS-detected Ca diffusion under vacuum conditions (essentially, zero humidity), in its limited extent, proves the availability of diffusion channels and their successful activation under electric fields. On the other hand, it also manifests the critical assistance that water molecules can provide in enabling the diffusion of large Ca quantities. The Ca ion (Ca^2+^), upon hopping from a specific egg-like cage to another, may be replaced by a water molecule, thus suppressing the buildup of a “stopping-field” under continued migration of ions.

For the composite material, long exposures to similar irradiation conditions, X-rays plus eFG, led to a similar effect on the Ca relative intensities (obviously referring to a smaller absolute Ca concentration because of the mixture with fibroin). However, in contrast to the development of a reduced spectral shoulder in pure pectin, a shoulder at a higher binding energy (see Figure 5b) could be observed for the Ca 2p doublet of the composite material, associated with more oxidized Ca. The latter observations point to interactions of the Ca atoms with the counter constituents of the composite complex (Figure 5c), presumably the carboxylic end-groups of fibroin or the water molecules. The pectin domains, on the order of 50 nm in average size, are large enough with respect to the XPS depth sensitivity so that a significant expression of the diffusion process can be realized in XPS intensities, even if the diffusion is limited to remain domain-restricted. Consequently, the field stabilized by the eFG was sufficient to enable a Ca diffusion efficiency comparable to that of pure pectin. However, the restriction of the Ca ions (Ca^2+^) to their original pectin domain is suggested by the absence of Ca reduction and, instead, the emergence of oxidation, in contrast to the pure pectin data. Eventually, diffused Ca may terminate its hopping at interfacial regions, where chemical oxidative processes took place. The supporting electrical measurements (see the SI) further reveal a considerable role of humidity in enabling interdomain Ca^2+^ diffusion, which can be distinguished from its intradomain diffusion. Complementarily, the XPS C 1s and O 1s spectra of pure pectin without Ca ions (Ca^2+^) are presented in Figure S17.

### Mechanical Properties of the Composite Biomaterial

**Figure 6:**
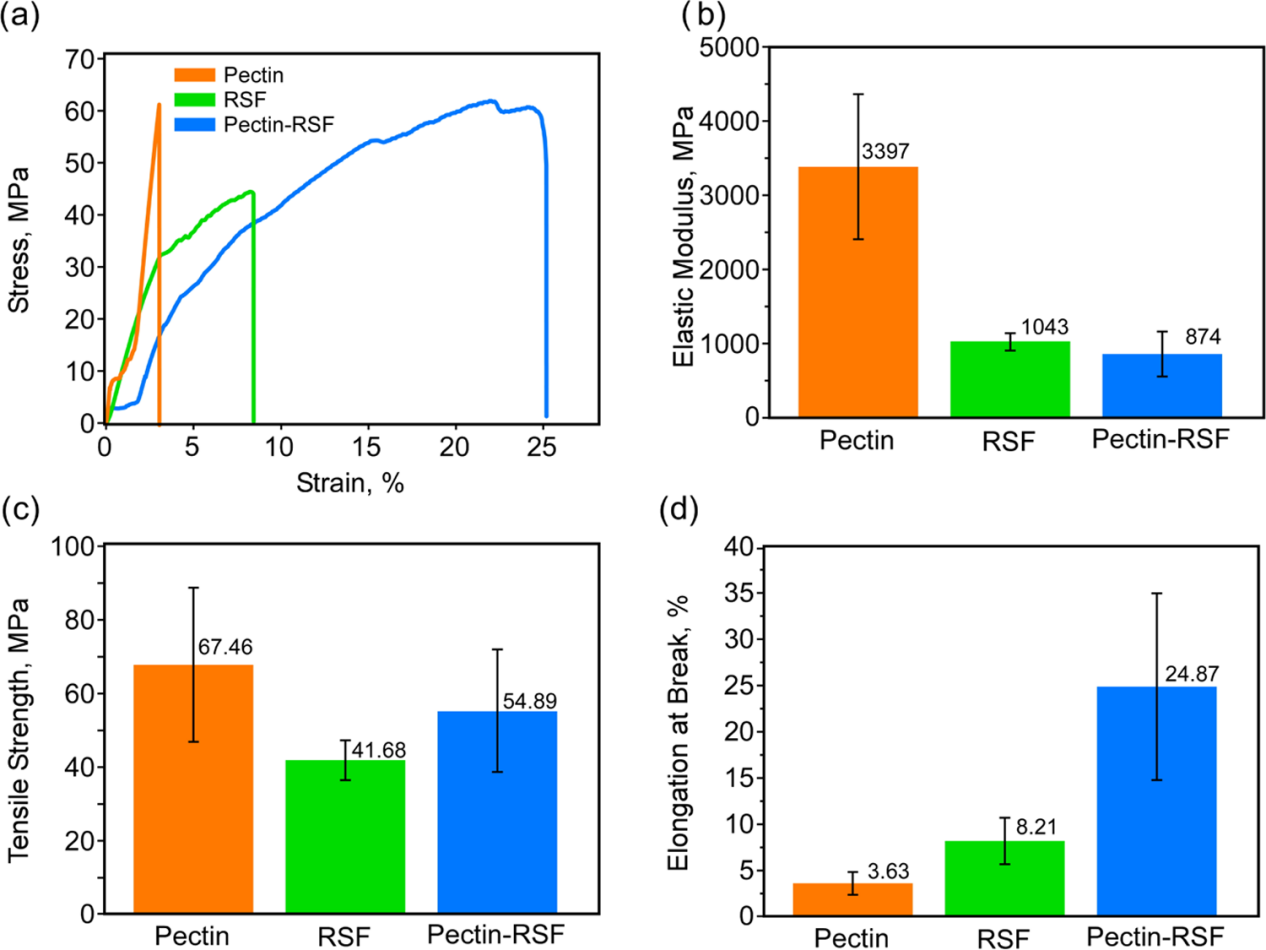
(a) Representative stress–strain curves for pectin, RSF, and the composite. Average and standard deviation values of the (b) elastic modulus, (c) tensile strength, and (d) elongation at break.

Finally, since the water content in silk materials is known to affect their strength, strain, and elastic modulus,^49^ we also analyzed selected mechanical properties of the one-component and of the pectin–fibroin composite films. Specifically, we analyzed the response of various films to uniaxial tension, shown in Figures 6 and S18a–c. Interestingly, the elastic modulus of the one-component pectin film was observed to be significantly higher than that of the RSF one-component film, which likely originated from the differences in internal structural organization. Thus, pectin spontaneously forms highly ordered layered structures (see inset in Figure 1a), compared to the RSF disordered organization (see inset in Figure 1b). Notably, Figure 6a shows a marked difference between the pure materials and their composite, where pronounced plasticity is manifested in the latter, higher than that of pure pectin and fibroin. This result is a clear indication of the significant role of domain interfaces in the composite film. Elongation and plasticity most likely reflect interdomain displacements, allowed by the weak fibroin–pectin interactions. Specifically, the composite film exhibits an increased strain value, Figure 6a,d (24.87%; displacement-controlled), compared with the one-component materials (3.63% for pectin and 8.21% for RSF). The elastic modulus was calculated from the linear slope of the strain stress curve. In the case of two-step curves (slope-plateau and then again slope-plateau) in pectin and composite films, the first slope was considered as an elastic modulus. Consistent with the strain data, the elastic modulus and the tensile strength of the composite materials are slightly lower (elastic modulus 847 MPa), compared with the one-component silk films (elastic modulus 1043 MPa), but significantly higher than pectin-only films (elastic modulus of the first slope is 3397 MPa; see Figure 6a), as summarized in Figure 6b,c.

Owing to the presence of hydrophilic groups in their macromolecular chains, such as hydroxyl groups (−OH), the mechanical properties of biological materials are frequently sensitive to humidity. In the presence of water, the hydrophilic groups tend to create hydrogen bonds with water molecules instead of adjacent protein or polysaccharide molecules. Thus, both higher free volume and weaker physical interactions between the components are realized, resulting in degraded mechanical performance of the mechanical properties.^50^ The segregated phase separation between the biopolymers at the nanoscale, observed in our previous results, introduces an additional impact on the mechanical properties. The related lower strength and elastic modulus values in the composite are likely the result of poor adhesion between pectin and the protein, which gives rise to reduced stress transfer and higher strain values.

Even though all samples exhibit large variability in the measured stress–strain curves, pectin and the composite films fail progressively to accommodate larger strain values compared to RSF, as expressed by the inelastic region in their curves. In the case of pectin, stress oscillation is observed, apparently the result of gradual microfracturing of layers within the lamella-like structure, see Figure 1a.^51^ In contrast, the composite materials maintain a relatively constant maximum stress plateau, characteristic of damage tolerance such as the domain sliding along interfaces.^52^ Pectin exhibits a strain-hardening behavior, with gradual resistance to failure.^53^ This strain-hardening behavior, which is characteristic of polysaccharides,^54^ is preserved in the composite. Finally, we performed a set of mechanical performance experiments under two humidity conditions (30 and 95%). The results are summarized in Figures 6a–d and S18. As expected, in all samples, the elongation was increased (in pectin by ∼4-fold, in silk by ∼13-fold, and in composite by ∼2-fold), whereas the elastic modulus decreased under increased humidity (in pectin by ∼14-fold, in silk by ∼33-fold, and in composite by ∼4-fold). Relatedly, a decrease in strength (in pectin by ∼10-fold, in silk by ∼20-fold, and in composite by ∼4-fold) was observed. These results indicate the role played by both parameters, i.e., the humidity and the nanodomain structure in governing the mechanical performance of the biopolymeric material in the composite form. Thus, regarding the pure silk films, changes in humidity caused a huge variation in the mechanical performance of the material, enabled via unwinding protein chains, thus achieving 110% extensibility at 95% humidity (compared with 8% in the dry state). However, the formation of domains in composite material limits such variations, which makes the material more stable under high humidity conditions, compared with the one-component films (see Figure S18c: the elastic modulus of pectin at 95% humidity is 244, that of RSF is 33.75, and that of pectin–RSF is 247.7 MPa).

## Conclusions

In summary, we analyzed the structure–property relationships in protein–polysaccharide composite materials obtained by combining pectin molecules and silk fibroin. The newly formed composite pectin–silk material is characterized by increased hydrophilicity and water-holding capacity as well as by improved mechanical extensibility and enhanced sensitivity of the ionic conductance to changes in humidity and temperature. These material characteristics emerge from a segregative type of assembly and the formation of protein- and polysaccharide-rich nanoscale domains. The nanoscale domains dictate conformational changes in silk protein, including reorientation of its repetitive hydrophilic sequences toward domain interfaces. Yet most influential is the introduction of plural domain interfaces. These domain interfaces introduce efficient water trappers, thus affecting the hydration of the composite material, its mechanical strain, and its ionic conductance. These results present a markedly nonlinear combination of the original constituent properties, believed to underlie a variety of evolution-selected sugar-protein-based biological systems. Moreover, a promising platform is proposed here for programmed, interfacial-based integration of biopolymers in modular multifunctional composite materials.

## Experimental Section

### Materials

Pectin solution was prepared from the commercially available beetroot low-methoxylated pectin with 34% methylation and 84% galacturonic acid (Herbstreith & Fox).

Silk fibers spun by the larvae of the silk moth *Bombyx mori* were degummed according to an established protocol.^55^ Briefly, silkworm cocoons were chopped and then boiled in 20 mM sodium carbonate solution (≥99.5%, Fisher Chemical, USA) at a ratio of 200 mL solution per gram of raw cocoon.^55^ The degummed fibers were then washed and dried, followed by dissolution at 60 °C in a concentrated solution of aqueous lithium bromide (≥99%, ReagentPlus, Sigma-Aldrich, USA; mass ratio 4:5 LiBr/H_2_O) at a concentration of 100 mg/mL. The resulting solution was centrifuged and dialyzed against Milli-Q water over 48 h using a 10 kDa cutoff membrane (Snakeskin, Thermo Fisher), followed by lowering the pH to 7 by using a solution of monosodium phosphate (≥98%, BioReagent, for molecular biology, anhydrous, Sigma-Aldrich, USA). In all subsequent experiments, Milli-Q double-distilled water (DDW) was used.

### Preparation of the Pectin, Silk Fibroin, and Composite Thin Films

#### Fabrication of Pectin Thin Films

Pectin powder (1%, w/v) was dissolved in DDW at 60 °C and stirred at 600 rpm until a uniform solution was obtained. The solution was then degassed at 25 mbar for 2 h. Next, 3 mL of the pectin solution was poured into a 2 cm × 4 cm Perspex© mold, followed by solidification for 24 h. Then, 10 mL of 30 mM CaCl_2_ (≥99%, ACS Reagent, Sigma-Aldrich, USA) cross-linkers were added and maintained for 24 h to accomplish the cross-linking process, and then washed with DDW, to remove excess cross-linker. The films were then dried under ambient conditions, and their margins were removed, as shown in Figure S1a.

#### Fabrication of Silk Fibroin (RSF) Thin Films

First, 3 mL of 6% RSF monomeric solution (w/v), in a 2 cm × 4 cm Perspex© mold, and dried at 23 °C and 34% relative humidity (RH), were placed in a clean room for 48 h. Next, a water annealing procedure was applied by placing the film in a 25 mbar chamber under water-saturated conditions for 24 h. Then, the film was dried under ambient conditions for 2–4 h before a further examination took place, and its margins were removed, as shown in Figure S1b.

#### Fabrication of the Pectin–RSF Composite Thin Films

Equal volumes of fibroin and pectin solutions of 6 and 1% (w/v), respectively, were mixed for 2 h with a magnetic stir bar at 80 rpm. Then, 3 mL of the mixture was poured into the 2 cm × 4 cm Perspex mold and dried at 23 °C and 34% RH. The post-treatment annealing, followed by cross-linking, was performed under the same conditions mentioned in the sections above. Note that the doping of all films (pectin, silk, and the pectin–RSF composite) with Ca ions (Ca^2+^) does affect the conductivity since the amount of Ca ions (Ca^2+^) is correlated with the amount of pectin. Unbound Ca ions (Ca^2+^) are washed out from the films at the washing stage. Reference samples with different (higher and lower than 1:6) pectin/RSF concentration ratios were also prepared and tested (not discussed here).

### High-Resolution Scanning Electron Microscopy (HRSEM) Analysis

HRSEM images were obtained using Ultra-55 and SIGMA ultrahigh-resolution SEM systems (Carl Zeiss, Germany). The samples were placed onto aluminum stubs and fixed with carbon tape. The samples (thin films) were then partially coated with carbon paste, and 2–3 nm of iridium was sputtered using a CCU-010 HV high-vacuum sputter coater (Safematic, Switzerland) prior to imaging to improve the sample’s conductivity and image contrast.

### X-ray Photoelectron Spectroscopy (XPS) Analysis

First, 1 mL of each sample was spread on an HF etched, p-doped, 1 inch silicon wafer and then spin-coated at 3000 rpm for 60 s. XPS measurements were performed on a Kratos AXIS-Ultra DLD spectrometer, using a monochromatic Al Kα source at low power, 15–75 W, and detection pass energies of 20–80 eV. The pressure in the analysis chamber was kept below 1 × 10^–9^ Torr. An electron flood gun (eFG) was used to address the beam-induced charging effects and stabilize the surface potential. Then, the energy scale was corrected for surface charging effects by setting the C 1s peak to 285.0 eV, which was used as a convenient reference with no attempt to get an absolute scaling.^56^ The beam-induced damage effects were thoroughly investigated by performing repeated scans on a given spot in comparison to rapid scans at fresh spots. The stoichiometry changes upon long exposures are an example of exploiting these “damage” effects to learn about the diffusion of the Ca ions (Ca^2+^) under externally applied fields, as described in the main text.

### Fourier Transform Infrared (FTIR) Spectroscopy Analysis

The absorbance FTIR spectra (ATR) were monitored on a Nicolet iS50 single-beam FTIR (Thermo Fisher, USA) between 400 and 4000 cm^–1^, using the following machine settings: 32 scans/sample, Happ-Genzel Apodization, and 1 cm^–1^ spectra resolution. Films were placed in an open container at the same location of measurement for temperature and humidity equilibration (25 °C, 34% RH) for 3 h before sampling was made.

For a protein’s secondary structure comparative analysis, first, all spectra were normalized by a coefficient factor so that the Amide I peaks overlapped. This allowed the curve morphology to be studied visually for trends. Next, the region 1557–1732 cm^–1^ was closely examined for important locations in the second derivative using OriginPro 2019b (Origin Lab, Northampton, MA). The baseline between the two lowest points was obtained, and fixed peaks were assigned with low degrees of freedom at constant locations. Interpretation of these peaks followed these guidelines: 1610–1625 cm^–1^ for intermolecular β-sheet, 1935–1660 cm^–1^ for α-helix and random coil, 1665–1685 cm^–1^ for β-turn, and 1690–1705 cm^–1^ for antiparallel amyloid β-sheet.

### Rheological Analysis

Viscometer measurements were performed using cone and plate geometry (*d* = 40 mm, 0.5° cone angle) mounted on a stress-controlled rheometer Discovery HR-2 (TA Instruments, USA). Pectin samples were loaded onto the rheometer at 23 °C. The flow behavior of pectin was measured as a function of shear rate over a range of 5–2000 s^–1^ over 8 min with a time step of 2 s per measurement. Three replicas were used for each set of experiments. A solvent trap was placed on top of the geometry to prevent dehydration during the measurement.

### ζ-Potential

The surface charge of materials was measured using a Zetasizer Nano ZSP (Malvern Instruments, UK). Disposable folded capillary cells (CAT DTS1070) were used for measuring the ζ-potential. The experiments were performed at room temperature (25 °C) with an equilibration time of 25 s. Each sample was tested three times, with 100 runs per single measurement.

### Contact Angle Measurements

The sessile drop method is measured by a contact angle goniometer (Rame-Hart 100–00 230 NRL C.A.), using a microscope optical system to capture the profile of a pure liquid on a solid substrate. The droplet was deposited using a syringe pointed vertically down on the sample surface, and the camera captured the image, which was later analyzed using ImageJ analysis software.

### Atomic Force Microscopy (AFM) Imaging

A film sample was deposited on a clean glass slide. The sample was imaged on an AFM JPK Nano Wizard 4 (Germany). The images were processed with JPK data-processing software.

### Thermogravimetric Analysis (TGA)

The thermal stability of the fibroin, pectin, and pectin–fibroin-mixed films was determined using nonisothermal TGA, using an SDT Q600 instrument (TA Instruments, USA) in a translucent alumina 90 μL crucible. For TGA, 3 mg of film was heated at a rate of 10 °C min^–1^ under a nitrogen atmosphere over the temperature range of 25–800 °C, to assess the temperature of the maximum decomposition rate (*T*_d_^max^). Water loss was measured as the weight degraded after the first slope. All other major peaks were assigned via a derivative of the weight loss. The first derivatives of the TG curves were calculated automatically using TA Universal Analysis software and then verified using OriginPro 2019b software, applying a fast Fourier transform (FFT) smoothing of 20–50 points.

### Preparation of Electrodes for Thermal Measurements

Lithographic methods were used for electrode preparation on a surface for thermal and humidity measurements. Initially, the substrates of standard microscope glass (25 mm × 75 mm) or silicon wafer (p-doped, 400 μm thickness, with 280 nm of SiO_2_ layer, prime grade, University Wafer, USA) were first cleaned in a beaker with a Teflon glass holder containing freshly prepared “Piranha” solution (H_2_O_2_–H_2_SO_4_, 1:3 by volume. *Caution! Piranha solution is aggressive compound, handle it with care*) for 1 h. Subsequently, the slides were washed three times with deionized water, three times with TDW, and then once with isopropanol. In between the cleaning procedures, the slides were sonicated for 5 min in an ultrasonic bath DU-32 (Argo-Lab, Italy). After the cleaning procedures, the substrates were dried under a N_2_ stream. Then, the substrates were placed on a hot plate (200 °C) for 5 min and cleaned with an oxygen plasma asher (Plasmod March, USA) for 5 min at 150 W and an oxygen flow of 4–5 sccm. Next, the positive photoresist of S1805 (Kayaku-Microchem, USA) was spread on the substrate and spin-coated (PWM32, Headway Research, 5 s at 500 rpm, acceleration at 100 rpm s^–1^, then 40 s at 5000 rpm, acceleration at 1000 rpm s^–1^), and then soft-baked at 110 °C for 1 min and allowed to cool down to room temperature to form 0.45 μm thick layer of photoresist. Then, the CAD design (Layout Editor; kLayout 0.26.7) of the future electrodes was uploaded to MicroWriter ML3 (Durham Magneto Optics, UK) and the substrates underwent exposition with the following parameters: magnification, *x*3, illumination wavelength, 395 nm, exposure, normal, photoresist sensitivity, (70 mJ cm^–1^) to reach an error in resolution of less than 5%. Then, the exposed substrates were developed with MF-319 developer (Microposit, USA) for 1 min with gentle agitation, rinsed with TDW, and dried with N_2_. Next, the substrates were subjected to deposition of 100 nm of iridium (1 s^–1^, 5 × 10^–5^ mbar, Safematic CCU-010 HV high-vacuum sputter coater, LabTech, UK) or gold with 5 nm of chromium as an adhesion layer (1 s^–1^, a vacuum of 1–4 × 10^–7^ Torr, ODEM evaporator, ODEM, Israel). Finally, the sputtered substrates underwent photoresist removal with acetone in a sonication bath until their full removal, then rinsed with IPA and TDW, and dried with N_2_.

### Probe Electrical Measurements

*I–V* and *I*(*t*)-time-dependent measurements (also related to approach 1, in which humidity measurements have been carried out) were acquired in the two-electrode configuration. The material film was first placed on a glass substrate with lithographically formed gold or iridium electrodes (100 nm thickness, 5 mm width, and 50 mm length; the procedure principle is described in detail in the Preparation of Electrodes for Thermal Measurementssection) having a gap of ca. 1–5 mm. Next, stainless steel plates (6 mm × 30 mm) coated with 100 nm of gold or iridium were applied on the sample to provide good contact with electrodes formed on glass. Where possible, the wires were connected to the plates via silver paint (SPI, USA). Then, the assembly was fixed with stainless steel clamps, and they were also attached to the glass with silver tape. A copper wire of needle electrodes was applied to the assembly and connected to a probe station (Janis ST-500-2, Quantum Design, UK). The connections and films were preliminarily checked with the AC/DC digital multimeter UNI-T 61B (China). The probe station was connected to a Keithley 4200A-CVIV Multi-Switch Source-Meter Unit (USA). The software provided by Keithley was used to control the measurements. Double- and triple-shielded cables with BNC connectors were used. The time-dependent series of film resistance (current) was carried out at 1 V with an interval of ca. 1–60 s between each point and a holding time of 0 s. *I*–*V* measurements were carried out between −5 and +5 V and from −1 to +1 V, and back to −5 and −1 V, respectively, with steps of 0.1 V per second. The temperature in the probe station chamber was controlled by a LakeShore 336 Temperature controller (USA) with a precision of ±0.001 K. Humidity was created by placing a hot water vapor supply inside the chamber, which allowed the chamber to reach up to 70% humidity. When this was not possible, the sample was put in a desiccator with a hot water supply and allowed to reach up to 95% humidity.

### Macroscale Mechanical Measurements

Stress–strain curves of the examined films were carried out by using an Instron 3345 Tester (Instron, Norwood, MA) equipped with a 100 Newton load cell at a stretching rate of 2 mm s^–1^. Measurements were done under ambient conditions at 30 °C and at two different humidity levels: 30% and 95% RH. Stress–strain curves were plotted and Young’s modulus was determined from the slope of the low-strain region.

### X-ray Diffraction (XRD)

XRD of the films was carried out in reflection geometry using a TTRAX III (Rigaku, Japan) theta–theta diffractometer with a rotating Cu anode operating at 50 kV and 200 mA. A bent graphite monochromator and a PMT detector were aligned in the diffracted beam, and θ/2θ scans were performed under specular conditions in the Bragg–Brentano mode with variable slits. The 2θ scanning range was 1–80° with a step size of 0.025° and a scan speed of 0.4 degrees per minute.

### Wide-Angle X-ray Scattering (WAXS)

WAXS measurements were carried out at the X-ray Powder Diffraction Lab at the Weizmann Institute of Science (WIS) on a SmartLab (Rugaku, Japan) diffractometer equipped with a 9 W rotation anode Cu tube and a HyPix-3000 two-dimensional detector with a beam stopper (about 2 mm diameter). A quasi-parallel X-ray beam was formed by a multilayer mirror (CBO attachment, Rigaku) and passed through a 0.3 mm pinhole and a 0.1 mm collimator; in addition, a 1 mm pinhole was placed before the sample. The distance to the detector was 27 mm. The beam size on the samples was 140 μm × 120 μm. Samples were measured for 5 h. Measurements were carried out under ambient conditions.

## Supporting Information Available

Schemes of film fabrication; full FT-IR spectra of the films; XPS spectra of the materials; rheological analysis of the liquid materials; ζ-potential measurements of the liquid materials; contact angle measurements; thermogravimetric analysis of the films; schemes of the electrical measurements; changes of the electrical properties of the films at different conditions of humidity and temperature; XPS spectra of pure pectin; and time-dependent XPS spectra of the films (PDF)
