# Supplementary material for: From Basic Principles of Protein–Polysaccharide Association to the Rational Design of Thermally Sensitive Materials
Source: ACS Appl Mater Interfaces. 2024 Feb 8;16(7):9210–23. doi: 10.1021/acsami.3c12926 (PMC10895586; doi:10.1021/acsami.3c12926)
Supplement: Supplementary file 1 — am3c12926_si_001.pdf [file am3c12926_si_001.pdf]

Supporting information

From basic principles of protein-polysaccharide association to the rational design of thermally sensitive materials

Asaf Rosenberg[1‡], Aleksei Solomonov[1‡], Hagai Cohen[2], Dror Eliaz[1], Israel Kellersztein[3], Ori Brookstein[1], Anna Kozell[1], Linghui Wang[3], Hanoch Daniel Wagner[1], Chiara Daraio[3], and Ulyana Shimanovich[1]*

[1]Department of Molecular Chemistry and Materials Science, Faculty of Chemistry, Weizmann Institute of Science, Rehovot 7610001, Israel

[2]Department of Chemical Research Support, Weizmann Institute of Science, Rehovot 7610001, Israel

[3]Division of Engineering and Applied Science, California Institute of Technology, Pasadena, CA 91125, USA

Correspondence

*Dr. Ulyana Shimanovich, Department of Molecular Chemistry and Materials Science, Weizmann Institute of Science, 7610001 Rehovot, Israel

Email: ulyana.shimanovich@weizmann.ac.il

[‡]These authors contributed equally.

Supporting Figure S1

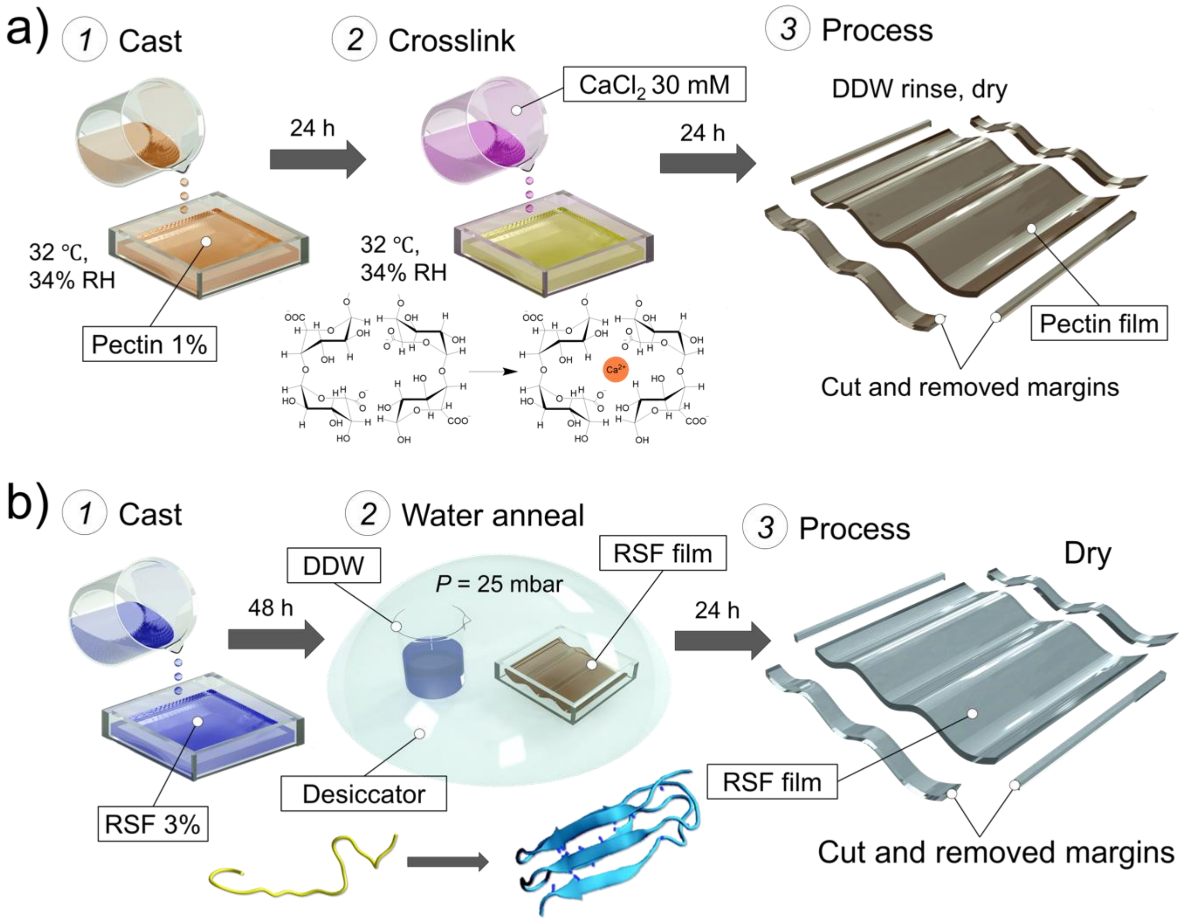

Figure S1. *Schematic representation of the detailed experimental procedure for thin film fabrication made of (a) pectin polysaccharide and (b) reconstituted silk fibroin (RSF) protein. Images were created with Autodesk AutoCAD.*

Supporting Figure S2

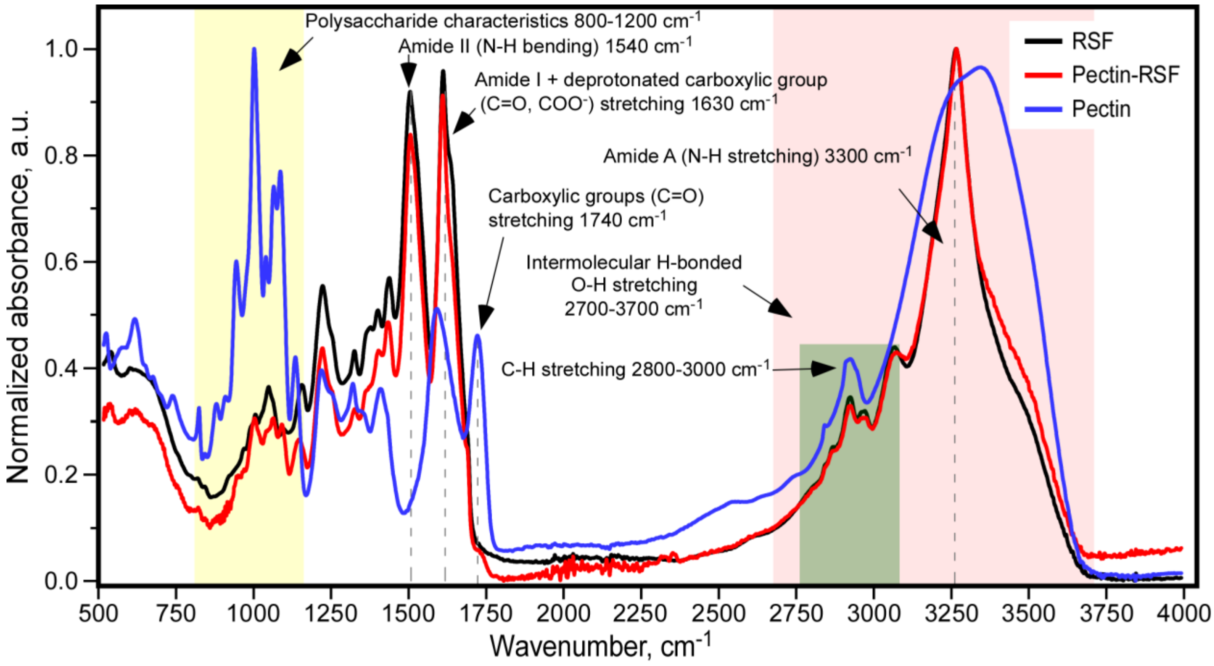

Figure S2. *Full FTIR spectra of the RSF film, pectin film, and pectin-fibroin composite film.*

Supporting Figure 3

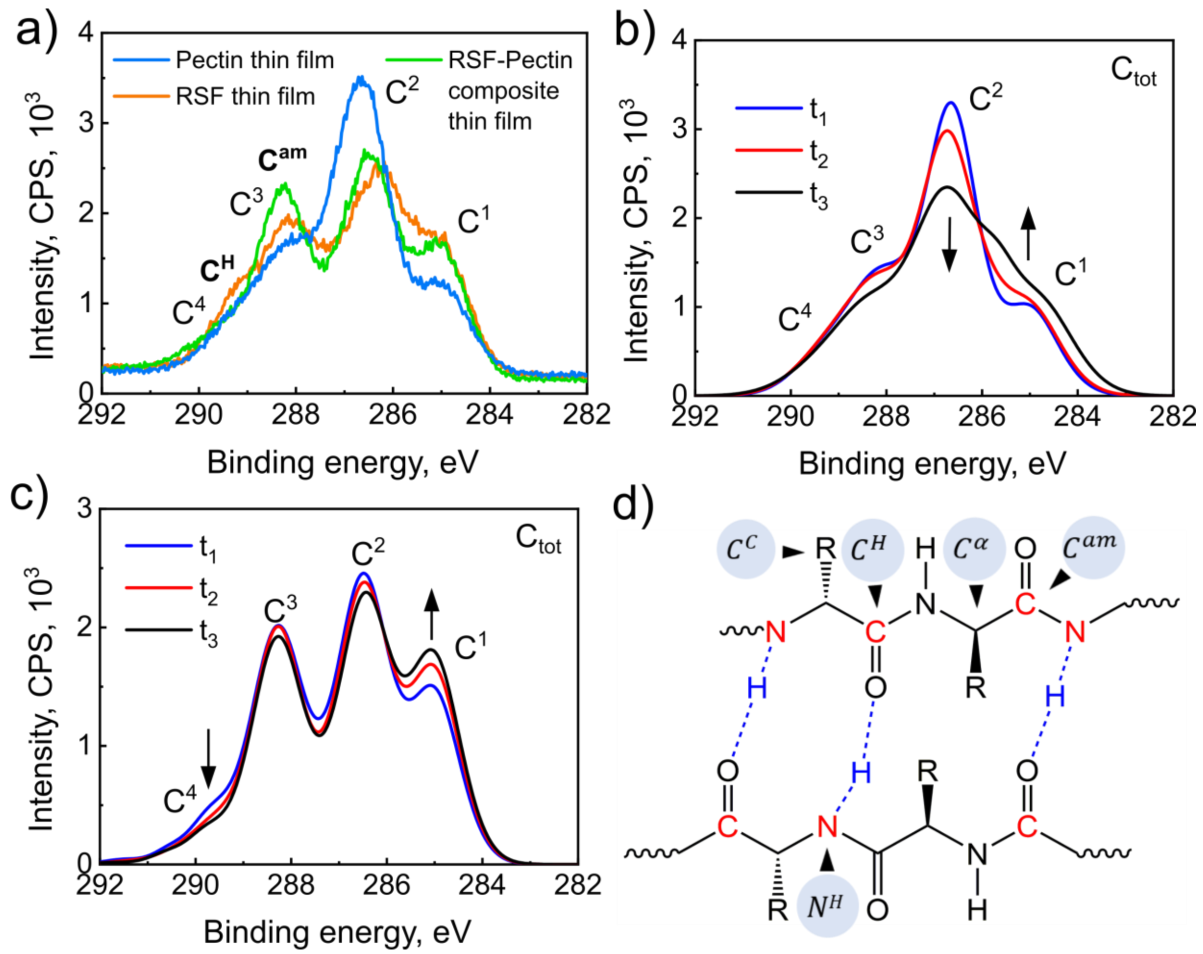

Figure S3. *Spectral details derived from X-ray photoelectron spectroscopy (XPS). (a) The C 1s XPS line of three different films: pectin (blue), silk fibroin (orange), and pectin-RSF composite (green). Beam-induced spectral changes in the C 1s line of (b) a pectin thin film; and (c) a composite thin film; (d) a schematic representation of the C and N states in the protein backbone. The labelling used for C components is consistent with our previous report [1], where C^1 stands for C-C and C-H bonds of various types; C^H stands for those amide groups (C^{am}) that take part in hydrogen bond formation through the carbonyl (C=O) bond, and C^2, C,^{3} and C^4 represent additional oxidation states typical of the pectin and peptide systems.*

Quantitative XPS analysis. Whereas the standard XPS derivation of atomic concentrations was rather trivial, the curve fitting process and the detailed interpretation of the results in the composite material encountered difficulties, due to (*1*) the presence of contaminating hydrocarbons and possibly water molecules; (*2*) residual charging-related spectral distortions; (*3*) beam-induced irreversible sample modifications that gradually occurred. The most reliable

encore in this analysis was found to be the protein-related concentration ratio between C amide (C^{am}) and the total N content – a characteristic fingerprint of the amino acid units in silk protein and, also, a feature relatively easy to interpret, recalling the absence of N content in the polysaccharide. As already shown in previous reports [1], for pure RSF samples, we expect a 1:1 C^{am}:N ratio, as indeed obtained very accurately, provided that both C^{H} and C^{am} were included.

The analysis of pure pectin proposes another useful concentration ratio, related to the composition of the Ca-filled cages. If each of the two non-methyl-esterified galacturonic acid rings in the polysaccharide chain are crosslinked with one Ca ion (Ca^{2+}), it means that in theory, for pure pectin samples, the Ca^{2+}/COOH ratio is supposed to be ≤ 0.5. Experimentally, a resolved shoulder appears in the O 1s XPS line, peaking at 531.5 eV (see **Figure 5c** in the manuscript), which is attributed to C-O-Ca in the filled cages. The intensity of this component yields a factor of ~4 in concentration ratios, compared to Ca, in agreement with the theoretical expectation. More accurately, this result suggests that 96% of the COOH groups are crosslinked (initially) to Ca ions (Ca^{2+}). Consistent with the former, the intensity of the COO component in the C 1s line of pure pectin, with a shoulder that peaks at 289.3 eV, gives the expected factor of 2 with respect to the amount of Ca in the pure pectin.

As a reference, we also provide spectra recorded from ‘Ca-free’ pectin, **Figure S16**. The low-binding-energy component in the O 1s spectrum is attributed to the carboxylic C=O bond, whereas its related C-O-H component appears at the high-binding-energy side. Notably, these signals are in good quantitative agreement with the carboxylic carbon component (see **Figure S3**).* These signals further present a quantitative difference compared with the samples incorporated with Ca (see a further discussion below), where both carboxylic oxygen atoms

* Note that, due to an overlap of components, a finite range of curve-fitting parameters yields equally high fit qualities. Hence, the significance of the present deconvolution is somewhat limited.

become essentially identical, giving rise to a doubly intense shoulder at the low-binding-energy side of the O 1s signal (see **Figure 5c** in the manuscript).

For the composite analysis, an overlap of a pectin signal with the C^{am} signal was encountered (see **Figure S3a**). Therefore, we used our fibroin reference, which has a 1:1 ratio between nitrogen and C^{am}, as an indicator of the fibroin content in the composite, relying on the measured N signals, and employed this result to the curve fitting process. Independently, the pectin amount in the composite could be estimated from the measured Ca signal, relying on its atomic concentration in the reference (pure) pectin samples. Thus, we could cross-check the reliability of our C 1s curve fitting by essentially relying on the N and Ca signals. Good agreement was thus obtained, indicating that the relative amount of Ca in the pectin domains was practically preserved upon composite preparation and also that the protein amide groups were preserved in this process.

Spectral XPS changes with time are shown in **Figures S3b,c**, reflecting the beam-induced degradation, mainly in the OH content, a result inferred from the combined elemental data. The degradation was particularly significant in the pectin sample, **Figure 5a** in the manuscript and **Figure S3b**, for which the backbone appeared to be of lower robustness. Accordingly, some increase in the non-oxidized C signal is seen in both **Figure S3b** and **Figure S3c**, attributed to *e.g.*, C-OH sites that 'lost' their OH groups. Analysis of these irreversible changes was mainly aimed at gaining confidence regarding our interpretation of the Ca signal and the evaluation of its ionic diffusion under externally applied fields (see the main text).

Evaluation of H-bond formation by using XPS. Technically, the XPS evaluation of changes in the H-bonded network is challenging. Hydrogen is commonly considered the only element that cannot be detected/quantified by XPS. However, based on changes in the binding energies of the neighboring atoms, associated with charge transfer upon H-bond formation, we have demonstrated in an earlier work the feasibility of achieving a reasonable accuracy by using this

analytical approach, applied to silk-based materials [1]. The dominant scheme of H-bond formation in β-sheet-rich proteins, such as silk fibroin, involves a backbone carbonyl and an amide nitrogen. Electron density around the O–H bond (in polysaccharide pectin and also in silk fibroin) is expected to increase upon the H-bond formation, on behalf of its neighboring sites: the nitrogen and the carbon. Accordingly, deconvolution of the XPS C 1s spectrum was found to yield four peaks of interest. The major ones in silk fibroin, labelled C^{C}, C^{α}, and C^{am}, correspond to CH_2/CH_3, α-carbon, and the amide carbons, respectively. The fourth component, C^{H}, is a shoulder to C^{am} and is associated with amide carbons that take part in H-bond formation. Unfortunately, this peak is not quite resolvable from carboxylic carbon that is present in pectin, as well as in one of the end groups of the fibroin chain. Therefore, unless the latter groups can be quantified by independent means, the H-bond quantification in composite samples becomes highly limited. However, this quantification can be strengthened by incorporating complementary information from the N 1s signal, seeking components of higher oxidation states due to the involvement of N in the H-bond on the other side of the hydrogen atom. Frequently, the N-atom belongs to a neighboring chain in the β-strand/β-sheet (see **Figure S3d**). Representative XPS spectra with indications for the C^{H} and C^{am} components are shown in supporting **Figure S3a**. A chemical shift of nitrogen (N^{H}) is also observed (not shown here) as a shoulder at the high binding energy side of the major N 1s line, and it is in good agreement with the above-described carbonyl oxygen of amides in the neighboring polymer backbones.

Given the uncertainty introduced by pectin signals that overlap with the C^{am}-C^{H} spectral range, our XPS-based evaluation of H-bonds in the mixed phase, and any changes in their amount, compared to pure RSF, cannot be considered sufficiently conclusive. Yet, with this reservation taken into account, our analysis suggests that a higher fraction of H-bonds is formed in the composite material, compared with both silk fibroin (RSF) only and pectin only

materials. This indication is further supported by our IR spectra, where an increased signal of *β*-sheets is seen, suggesting two possible explanations: *(1)* the competition between fibroin-fibroin, fibroin-solvent, and fibroin-pectin interactions triggers conformational changes *within* the silk constituents of the composite material, giving rise to a higher fraction of β-sheets, *(2)* H-bonds play a significant role specifically at the interface domains, namely, in the interactions *between* pectin and silk constituents.

Experimental details of the XPS measurements. Since the quantitative analysis of oxidation states is based on fine details in the line-shape of selected signals, it turns out to be very sensitive to *differential* charging effects and their related spectral distortions. This is an inherent artefact common to XPS of poorly conducting specimens. Therefore, minimization of the charging-related distortions was critical in this study. To address this issue, we used repeated scans under various charging conditions, controlled by the electron flood gun (eFG) parameters and the X-ray source power. In addition, differentiation of artefacts from the 'real' chemical information was improved by comparing the line-shapes of different signals, based on the assumption that, for any given location in the sample, the charging-related line-shifts would be the same for all signals. This approximation applies, in particular, for the line-shapes of C^{H} and C^{am}, for which we expect to obtain identical spectral distortions, thus enabling an improved derivation of the chemical information hidden in the former. Exceptions beyond this approximation, e.g., ones involving atomic-scale charge redistribution [2], were found to be of negligible relevance in the systems studied here. Combined with the repeated scans, work-function measurements [3] at reduced source power, <0.3 W, were applied, in order to better follow the time evolution of the surface potential. By performing repeated scans, we could also gain insight on the beam-induced sample modifications, an issue of general concern upon exposure of organic compounds to X-rays [4]. Thus, the effect of Ca-diffusion was revealed and evaluated here, as well as the beam-induced damage of the oxidized carbon moieties.

Supporting Figure S4

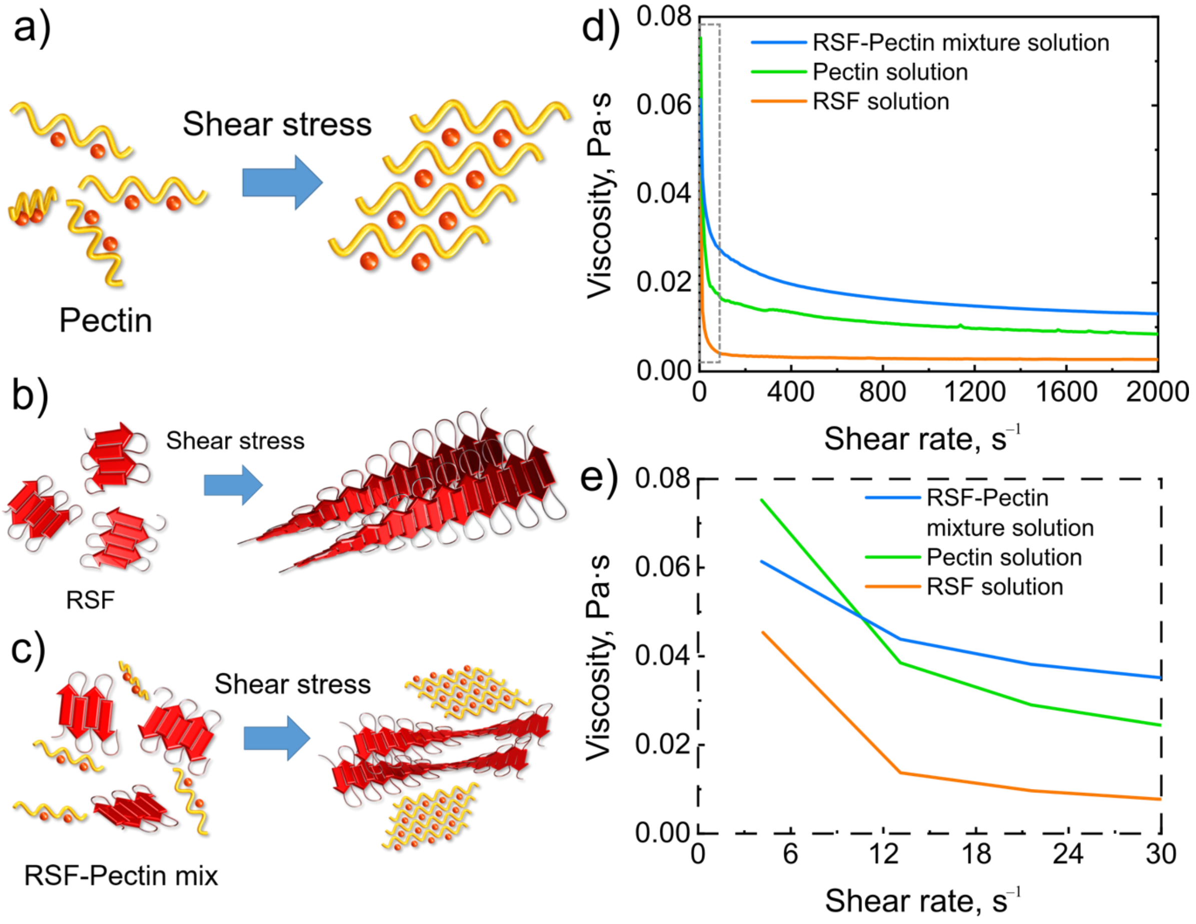

Figure S4. *Illustrations of the particle assembly in solution while applying shear force for pectin, 1% w/v (a), RSF, 3 % w/v (b), Pectin-RSF-mix, equal volumes of RSF and pectin solutions of 6% and 1% w/v (c). Images were created with Autodesk Fusion 360. (d) Rheological measurements showing the viscosity of the RSF solution, the pectin solution, and their mix. (e) Zoom-in of (d), showing the low shear rate values that reveal the difference in the shear thinning effect and that imply that the phase separation occurs within the mixture.*

Supporting Figure S5

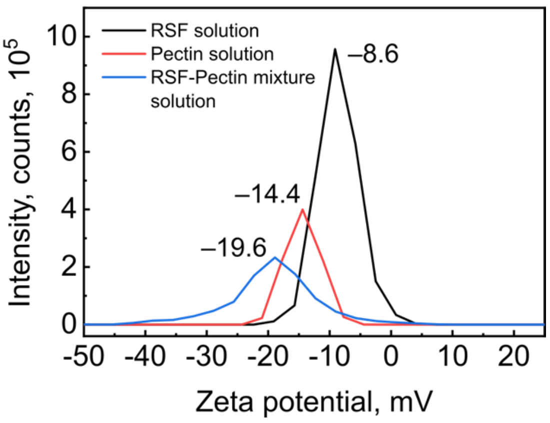

Figure S5. *Zeta-potential distribution for the systems of RSF (pH = 9.4, 3% w/v), Pectin (pH = 3.2, 1% w/v), and an RSF-Pectin mixture (pH = 4.6, equal volumes of RSF and pectin solutions of 6% and 1% w/v).*

Supporting Figure S6

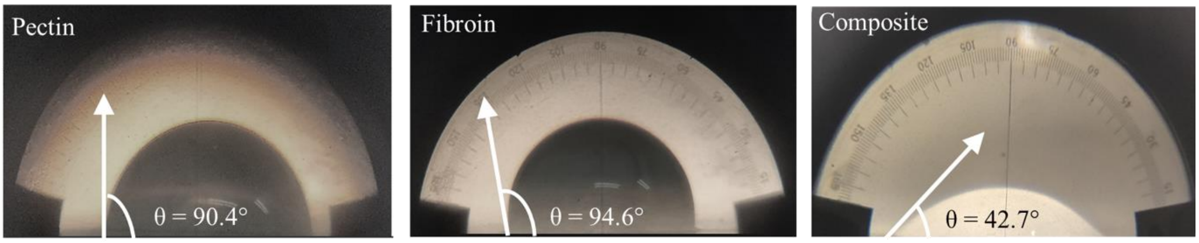

Figure S6. *Contact angle measurements for pectin, silk fibroin, and their composite.*

Supporting Figure S7

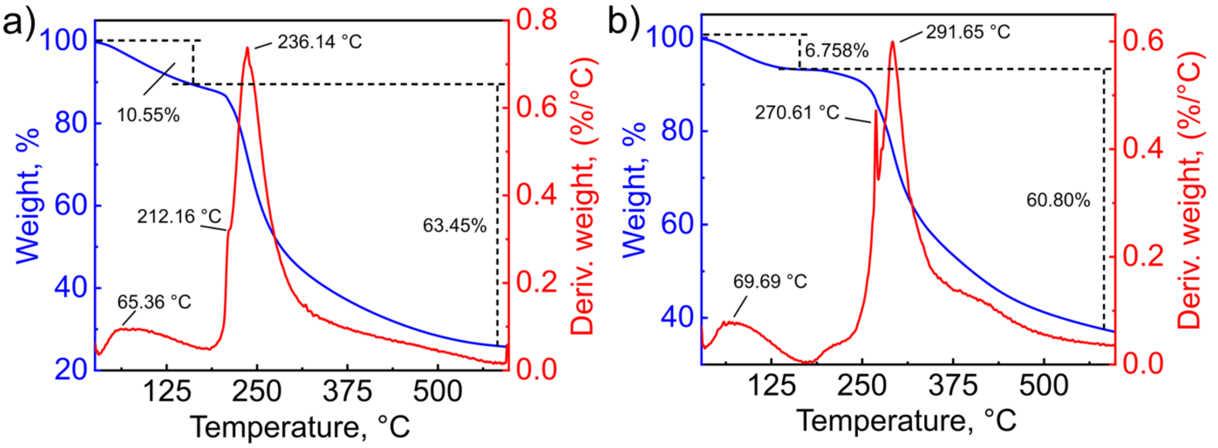

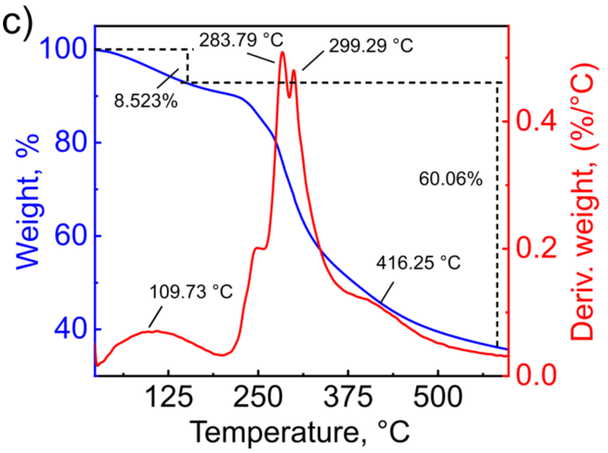

d)

Major peaks,°C

	Pectin	RSF	RSF-pectin mix	Assignment
1	65	70	110	Water molecules release
2	212	208	250	Intra-molecular hydrogen bonds break
3	236	279	283	Protein edges and saccharide backbone disassemble
4		291	300	Amorphous regions break
5		416	416	Crystalline regions break

Figure S7. *TGA (blue) and DTG (red) curves of the three synthetic films and their matching water loss mass percentage: (a) pectin film (water loss 10.55%); (b) RSF film (water loss 6.758%); (c) RSF-pectin-mixed film (water loss 8.523%); and (d) a table summarizing the measured temperature values for the corresponding films.*

Supporting Figure S8

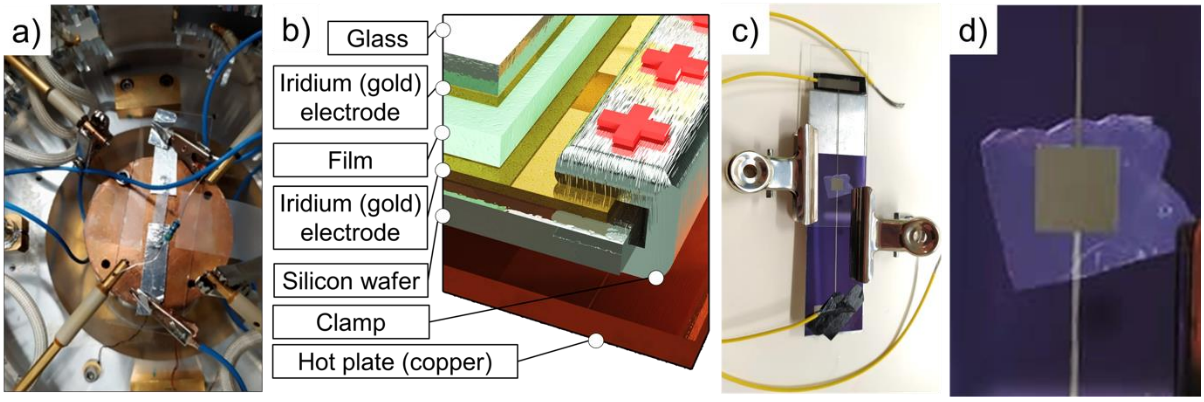

Figure S8. *(a) A sample of RSF–Pectin hybrid composite film of 60 µm thickness, 1 cm length, and 0.5 cm width fixed on a microscope cover glass with attached electrodes in the probe station chamber for humidity-varied studies. (b) A detailed scheme of the setup presented in Figure 4d (see main text) showing the measurement of the electrical properties of the films as a function of temperature. Image was created with Autodesk Autocad. (c, d) A sample of a Silk Fibroin–Pectin hybrid composite film of ~60 µm thickness fixed in between a pair of electrodes; the effective electrode area is 5x5 mm.*

Supporting Figure S9

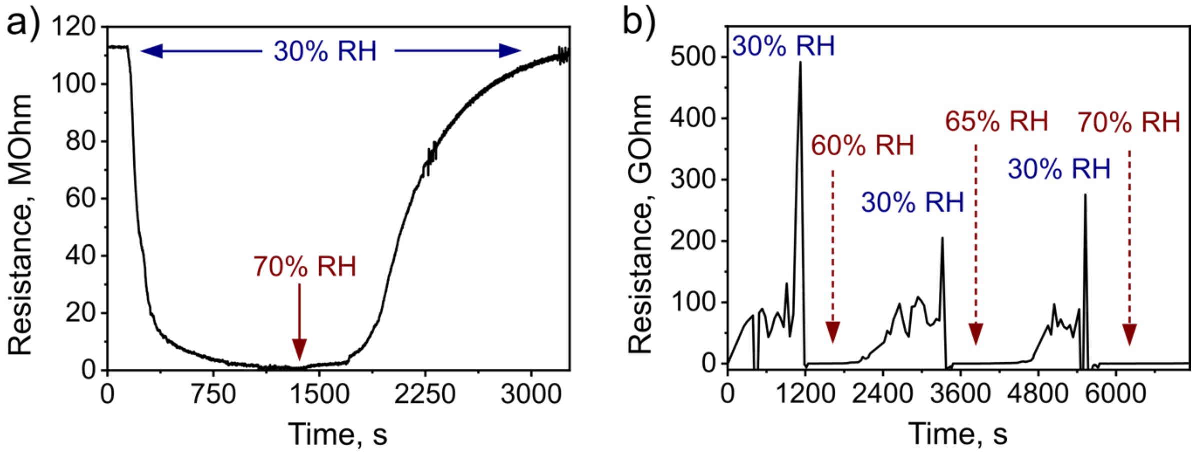

Figure S9. *(a) Changes in resistance as a function of the time of the pectin film at 23 °C, tested upon humidity variation from 30% to 70% and back to 30%; (b) changes in resistance upon humidity changes (humidity values 30, 60, 30, 65, 30, and 70%), measured at 20 °C for Silk Fibroin–Pectin hybrid composite film and plotted as a function of time. The applied voltage is 1 V. Resistivity of the film at 30% humidity and 20 °C is ca. 0.9 MOhm·m (11.1 nS·cm^{-1}), whereas at 70% the resistivity is ca. 20 KOhm·m (0.5 µS·cm^{-1}).*

Supporting Figure S10

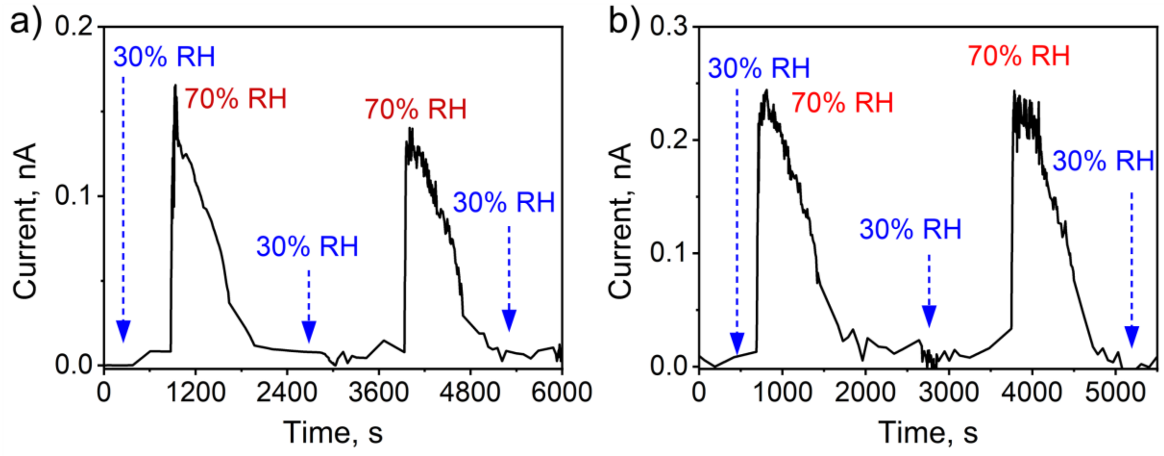

Figure S10. *Current changes in RSF (a) and RSF + Ca^{2+} (b) films as a function of time at 20°C, after applying changes in relative humidity. At the beginning, the relative humidity is 30%, then it is increased up to 70% and it gradually drops down to 30%. The second peak corresponds to a second cycle in humidity changes. The applied voltage is 1 V.*

Supporting Figure S11

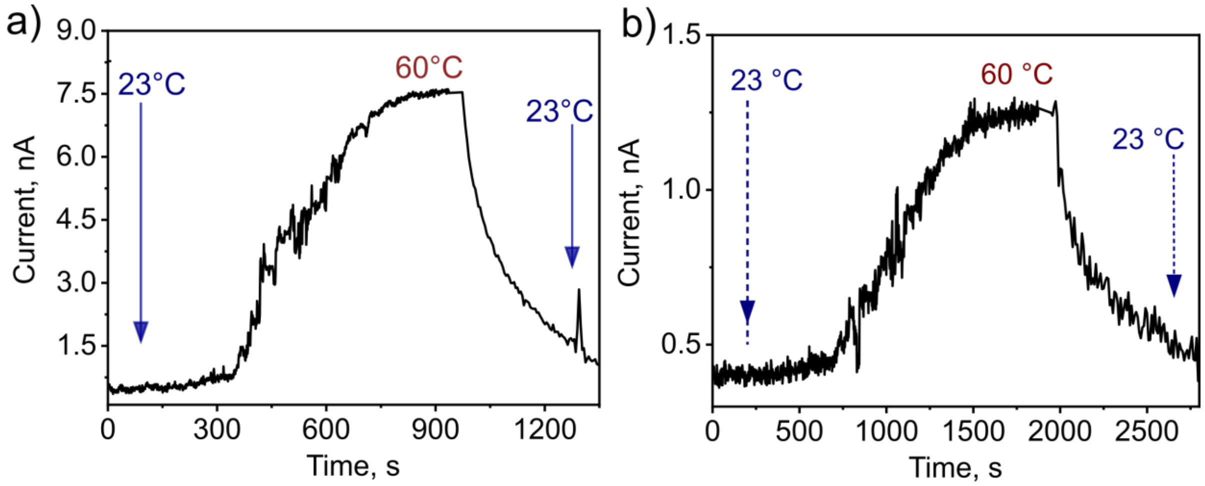

Figure S11. *Current changes in (a) RSF-Ca^{2+} and (b) RSF films (Ca-free) as a function of time upon elevating the temperature from 23 ℃ to 60 ℃ and then cooling down back to room temperature.*

Supporting Figure S12

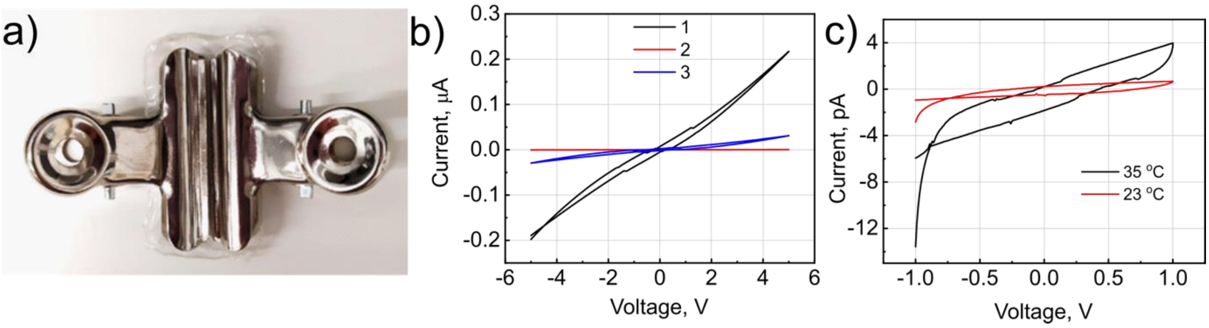

Figure S12. *(a) A photograph of the device with a pectin film, having a thickness of 10 um. The distance between clamp electrodes is 1 mm. The length of the effective part of the electrode is 38 mm; (b) cyclic voltammetry plots of the same film at 23 ℃ with (1), P = 1 atm, 70% relative humidity, (2) P = 1 atm, 43% relative humidity, and (3) P = 0.38 mbar (low vacuum conditions), 25% relative humidity; (c) Cyclic voltammetry at a relative humidity of 30% of the Silk Fibroin–Pectin hybrid composite film, recorded at different temperatures, 23 ℃ and 35 ℃.*

Supporting Figure S13

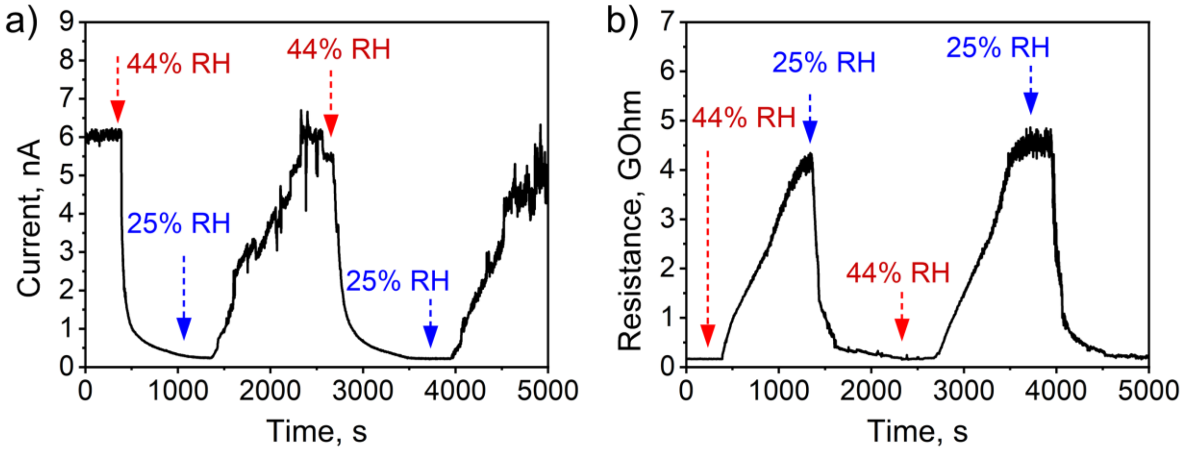

Figure S13. *Cyclic changes with the time of the resistance (a) and current (b) of a big pectin film piece at 23 °C, when humidity is decreased from 44% to 25% and back to 44%.*

Supporting Table S1

Conditions	Measured conductivity ($\mu S \cdot cm^{-1}$)			
	**Pectin (+ Ca^{2+})**	**RSF-Pectin Composite (+ Ca^{2+})**	**RSF**	**RSF (+ Ca^{2+})**
20-23 ℃, 30% humidity	0.15	0.011	0.0002	0.0004
20-23 ℃, 70% humidity	1.5	0.6	0.0014	0.0028
60 ℃, 40% humidity	17.6	0.95	0.0006	0.0046
Thermal potential	106.7 a.u.	73.7 a.u.	3.1 a.u.	14.5 a.u.

Table S1. *Conductance of the organic films at various conditions, based on* ***Figure 4*** *(the main paper) and* ***Figures S9-S13****.*

Supporting Figure S14

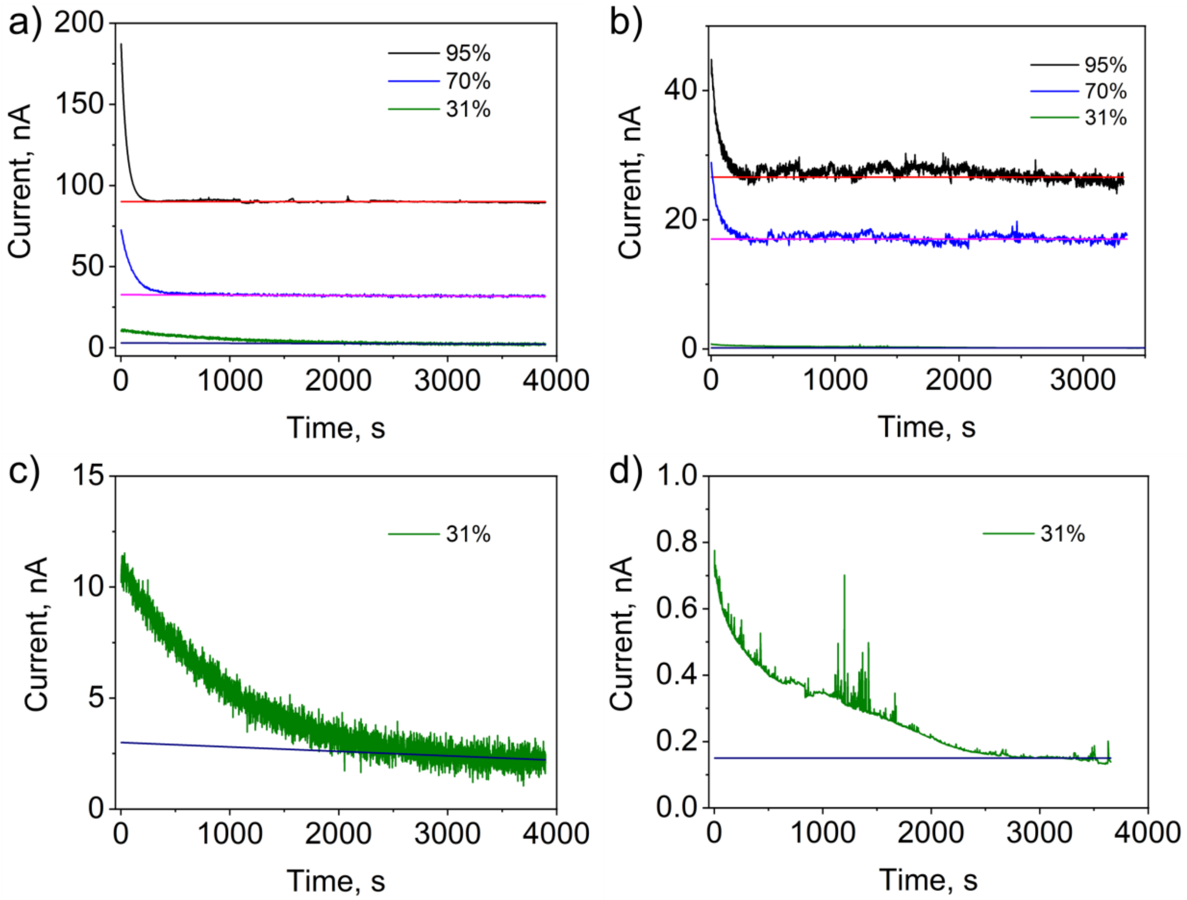

Figure S14. *Electrical measurements (current vs. time) under controlled humidity conditions of the (a) pectin-Ca^{2+} film and (b) Silk-Pectin-Ca^{2+} hybrid composite film (right), performed at 23 °C and with ambient humidity (RH%) values of ca. 95, 70, and 31%. Zoom into the early stages of the low humidity curves is provided in the bottom panels, (c) for the pectin and (d) for the composite films. The applied voltage is 1 V.*

Insight into the ionic conduction processes in the studied samples can be gained from our electrical measurements, see **Figure S14**, performed under controlled humidity and temperature conditions. All current curves in this figure, *I(t)*, present two distinct stages: a roughly exponential decay in *I(t)*, followed by a persistent constant current magnitude. We attribute the first stage to simultaneous conduction of Ca ions (Ca^{2+}) and protons (H^+) ions, whereas the second stage is interpreted as ‘only proton’ conduction. The proton conduction magnitude is expected to strongly depend on humidity, a fact manifested by the marked

increase in background (the constant current) value for each sample. In contrast, the Ca-ion conduction presents a capacitor-like discharge behavior, for which the *total amount* of charge ($Q=\int I(t)dt$) is a sample-specific constant feature, independent of humidity. Consequently, integration over time throughout the first stage in the three humidity curves of each sample should give the same Q-value for all humidity levels, provided that the background level is subtracted to get the net Ca-related charge that was transported across the device.

Experimentally, the latter feature is confirmed for the pure pectin sample, obtaining similar Q values (**Table S2**). Minor differences in the Q values of the pectin sample emerged from the chronological order of measurements, due to incomplete reversibility in the Ca-ions migration: The first scan was taken at 31%. It was followed by a re-charge step with reversed bias for ~10 minutes. The second scan, at 70%, was followed as well by a similar re-charge step and then, the third scan was performed at 95%.

Compared to the Q values of pure pectin, **Table S2** shows that the Q-values in the composite system are significantly smaller. Indeed, the nominal amount of pectin in the mixed phase is only 20-25%; hence, the available amount of Ca should similarly be reduced by a factor of 4-5. In addition, a mosaic distribution of pectin domains in the composite would suggest an interesting test of conductance across the RSF domains. By testing the reference RSF samples, with and without Ca content, we first verified that pure RSF is a very poor conductor for Ca ions (Ca^{2+}, also for protons), independent of the humidity level.[*] Therefore, also in the composite system, ion conductance across RSF domains is expected to be very poor. Indeed, under 31% humidity we received supportive results, consistent with a model in which the pectin domains are partially separated (with RSF domains in between). In other words, the Q-values here represent a small portion of the pectin domains, those located near the electrodes. On the other hand, under higher humidity levels, the ‘across RSF’ connectivity appears to

[*] In pure RSF current at saturation (I_{sat}). is: ca. 0.01–0.02 nA at 31% and ca. 0.13–0.16 at 70% (see Figure S10).

gradually improve, so that at sufficiently high levels (95%) the *Q*-value approaches the nominal factor (20-25% of the pure pectin) in Ca amounts. This result is in an apparent contradiction to the poor humidity dependence obtained with pure RSF. Consequently, these results indicate that pectin-RSF domain interfaces, at sufficiently high humidity, play a significant role in assisting the Ca conductance. An independent support of this result is given in the contact angle measurements, **Figure 3** (main text), and XPS-derived Ca migration, **Figure 5** (main text).

Note also that for the composite sample at 31% humidity, two resolved parts are seen in the first stage of the *I(t)* curve (**Figure S14d**). We attribute this split to the presence of pectin domains that are partially disconnected from the electrodes. In contrast to those domains that have reasonable connectivity directly to the electrodes, the ones located farther would encounter a significant delay in Ca migration under low-humidity conditions.

Finally, and most striking, **Figure S14** provides clear evidence for the cross-talk between the mechanisms of Ca ions (Ca^{2+}) and proton (H^{+}) conduction. The decay rate (*i.e.* the slope) in *I(t)* at the first stage of these curves exhibits high sensitivity to humidity, as is easily seen in **Figure S14**. It is manifested by marked differences in the time the system takes to approach the second stage. The values of the RC time constant of this capacitor-like system are given in **Table S2**, reflecting this feature. The latter effect is more pronounced in pure pectin, because in the composite the ‘resistant’ RSF domains act to suppress current for all humidity levels.

Supporting Table S2

Relative humidity, RH%	Ca-related charge[*] (Q, 10^{-6} Coulomb)		Current at the saturation point ($I_{sat.}$ nA)		RC (s)	
	Pectin + Ca^{2+}	Pectin-RSF composite + Ca^{2+}	Pectin + Ca^{2+}	Pectin-RSF composite + Ca^{2+}	Pectin + Ca^{2+}	Pectin-RSF composite + Ca^{2+}
31	7.6	0.27 (1st step) 0.46 (total, after 2nd step)	2.15	0.35 (1st step) 0.15 (total, after 2nd step)	980	270 (1st step) 345 (2nd step)
70	6.15	0.73	31.8	17.0	106	71
95	4.86	1.12	90.1	26.6	50	63

Table S2. *Characteristic electrical values in the pure pectin and the composite samples. Ca-related charge values (Q) are derived by first removing the background level and then integrating over time for each curve in* ***Figure S14****. The saturation current represents the proton-conductance magnitudes. The RC time constants were derived from the exponential decay (during the first stage in each plot) for the pectin and its hybrid composite with silk. Calculations were performed numerically in Origin 2021b.*

[*] The current decay under these conditions exhibited two resolved parts, here referred to as the 1st and 2nd. Accordingly, two different values are provided for the Ca-related charge, the saturation current, and the RC time constant.

Supporting Figure S15

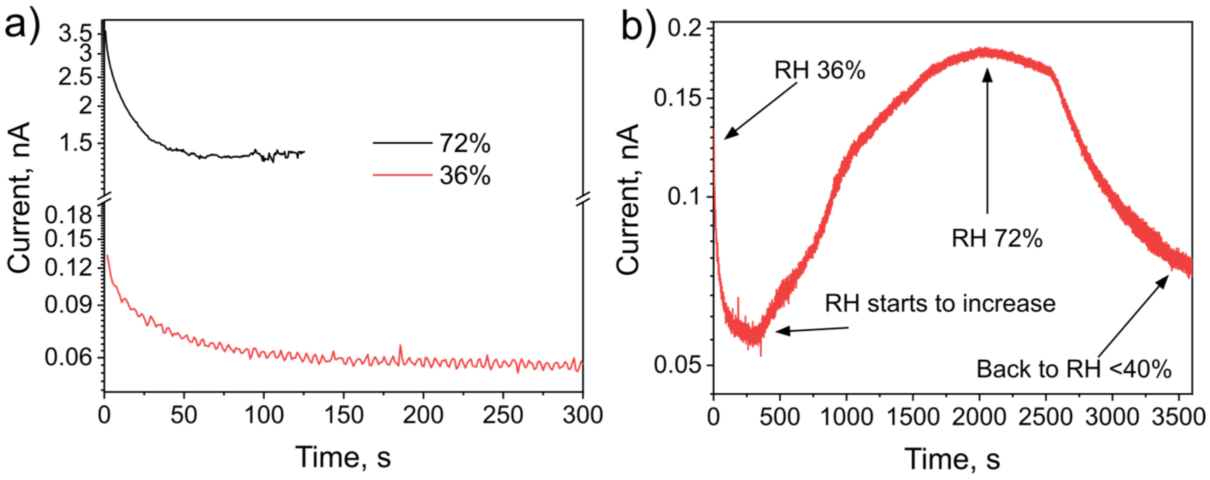

Figure S15. *Electrical measurements (current vs. time) under controlled humidity conditions of the RSF-pectin films without Ca ions (Ca^{2+}):(a) a comparison of the polarization curves at two values of humidity measured separately and (b) the current change in the same film upon exposure to gradual, smooth humidity changes.*

In our experiments, changes in current vs. time for undoped with Ca ions (Ca^{2+}) RSF-pectin films upon changing the relative humidity from 36% to 72% resulted in a ca. 22-times increase in conductivity. That effect is smaller than in the cross-linked film (see **Table S1**). Therefore, the presence of additional electrolyte ions influences the conductivity. Overall, the doping of all films (pectin, silk, and the pectin-RSF composite) with Ca ions (Ca^{2+}) affects the conductivity to some extent. Note that the amount of Ca ions (Ca^{2+}) is correlated with the amount of pectin. It is also important to note that unbound Ca ions (Ca^{2+}) is washed out of the films at the washing stage.

Supporting Figure S16

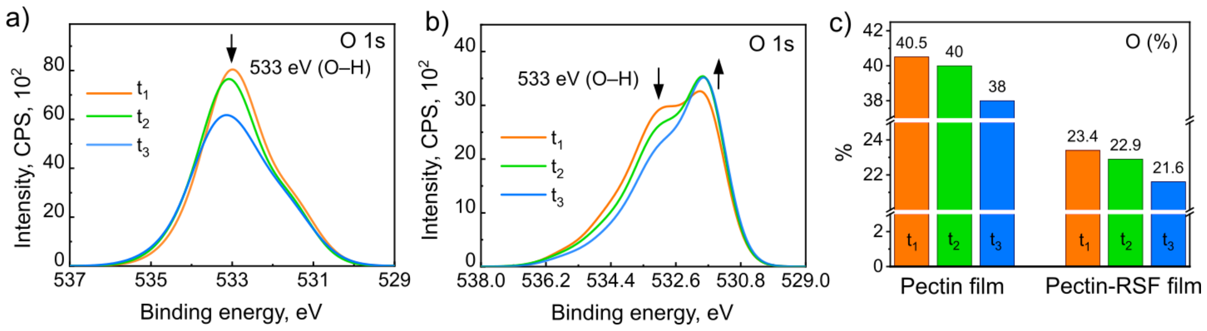

Figure S16. *(a-c) Time-dependent X-ray photoelectron spectroscopy (XPS) analysis of beam-induced changes, referring to three different times throughout the experiment, 12 (t_1), 60 (t_2), and 120 (t_3) min: (a) the oxygen (O 1s) line of a pectin-only film and (b) of a pectin-fibroin composite film; (c) a quantitative summary of the total oxygen intensities in the two systems, given in atomic % for t_1-t_3.*

Supporting Figure S17

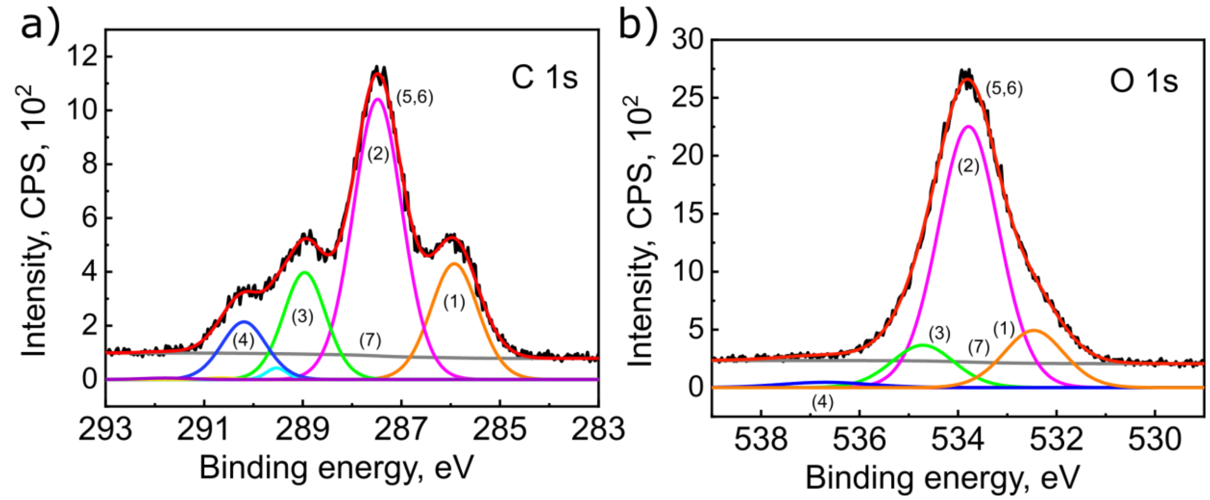

Figure S17. *Selected XPS lines of an uncross-linked pectin sample, free of* Ca^{2+} *ions: a) C 1s, where the specific curves stand for (1) C–C, C–H, (2) C–O, (3) C=O, (4) COO, (5,6) raw spectrum (black) and the calculated envelope (red), (7) background; b) the O 1s spectrum where the specific curves stand for (1) C=O, (2) C–O, (3) O–H, (4) background, (5,6) the raw spectrum (black) and the calculated envelope (red), and (7) background.*

Supporting Figure S18

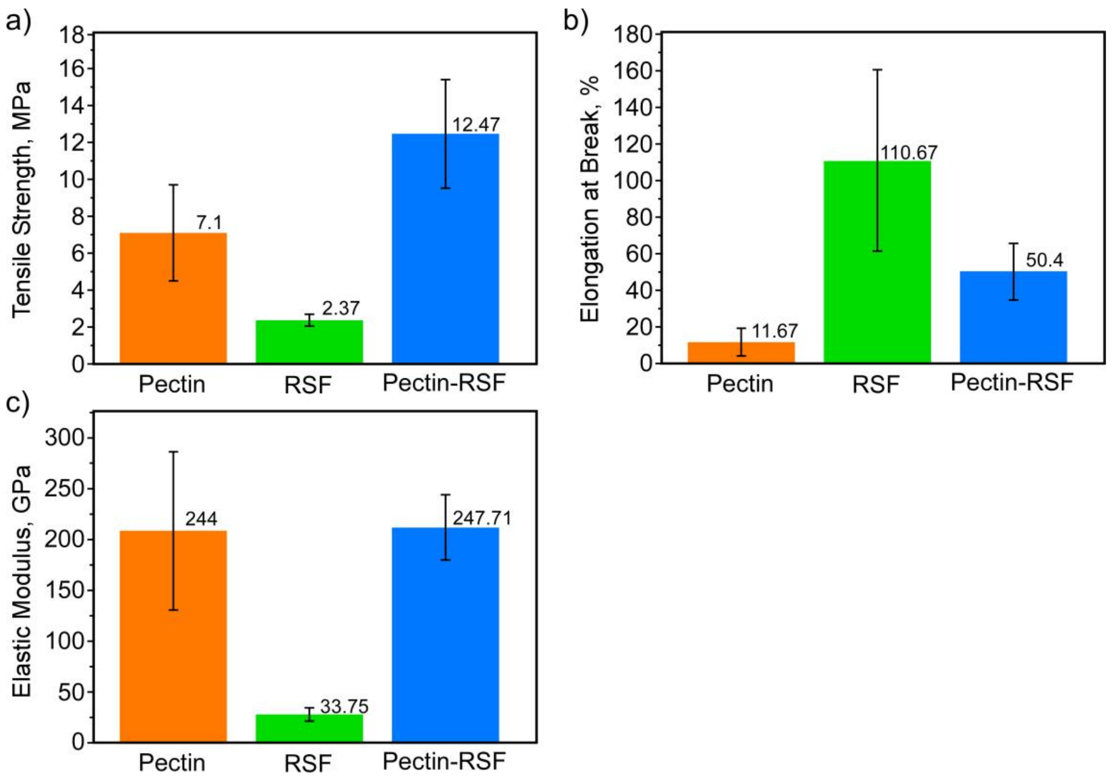

Figure S18. *Stress-strain curves for (a) pectin, (b) RSF, and (c) their composite, as extracted under 95% humidity. Representative stress-strain curves for pectin, RSF, and the composite. Average and standard deviation values for pectin, RSF, and the composite films evaluated under high humidity conditions (95%): (a) tensile strength; (b) elongation at break; (c) elastic modulus.*

References

1. Aggarwal, N. *et al.* Protein nanofibril design via manipulation of hydrogen bonds. *Commun. Chem.* **4**, 62 (2021).
2. Filip-Granit, N., Van Der Boom, M. E., Yerushalmi, R., Scherz, A. & Cohen, H. Submolecular potential profiling across organic monolayers. *Nano Lett.* **6**, 2848–2851 (2006).
3. Itzhaik, Y., Hodes, G. & Cohen, H. Band alignment and internal field mapping in solar cells. *J. Phys. Chem. Lett.* **2**, 2872–2876 (2011).
4. Frydman, E., Cohen, H., Maoz, R. & Sagiv, J. Monolayer damage in XPS measurements as evaluated by independent methods. *Langmuir* **13**, 5089–5106 (1997).
